# KRAS Inhibitor–Induced Cancer Cell Death Enhances Sensitivity to Immune-Mediated Bystander Killing of Drug-Resistant Subclones

**DOI:** 10.1158/0008-5472.CAN-25-5693

**Published:** 2026-06-08

**Authors:** Mona Tomaschko, KangBo Ng, Christopher Moore, Claire E. Pillsbury, Sareena Rana, James Campbell, Saptaparna Mukherjee, Anna Mikolajczak, Panayiotis Anastasiou, Andrea de Castro, Alicia Alonso de la Vega, Sophie de Carné Trécesson, Nathan W. Goehring, Miriam Molina-Arcas, Julian Downward

**Affiliations:** 1 Oncogene Biology Laboratory, The Francis Crick Institute, London, United Kingdom.; 2 Polarity and Patterning Networks Laboratory, The Francis Crick Institute, London, United Kingdom.; 3 Bioinformatics and Biostatistics, The Francis Crick Institute, London, United Kingdom.; 4 Experimental Histopathology, The Francis Crick Institute, London, United Kingdom.

## Abstract

The clinical benefit of therapies targeting the *KRAS* G12C mutation is substantially limited by the development of resistance through multiple mechanisms. Therefore, to achieve lasting benefit with these therapies, effective strategies to tackle the evolution of drug resistance are required. To investigate this, we developed a preclinical model that mimics the development of resistance to KRAS G12C inhibitors (G12Ci), such as adagrasib and RMC-4998. Treatment of tumors containing a minor subpopulation of resistant cancer cells with G12Ci led to their rapid outgrowth, replacing drug-sensitive cells within a few weeks. However, combining G12Ci with therapies that enhance immune responses, such as anti–PD-1 or SHP2 inhibitors, resulted in the elimination of resistant cells and durable complete responses, even though anti-PD-1 or SHP2 inhibitor monotherapy did not induce regression of resistant tumors in the absence of drug-sensitive cells. This bystander killing of resistant cells following targeting of drug-sensitive cells was dependent on an intact adaptive immune system. Mechanistically, these combination therapies led to profound remodeling of the tumor immune microenvironment, making it less immunosuppressive, and promoted cancer cell death that primed an immune response, with an influx of cytotoxic T lymphocytes recognizing tumor-associated antigens shared between G12Ci-resistant and -sensitive cancer cells. Promotion of immune-mediated bystander elimination of drug-resistant cells may provide a paradigm for tackling the problem of drug resistance in cancer more broadly.

**Significance:**

Combination therapies that stimulate antitumor immunity promote immune-mediated bystander killing of KRAS-G12C inhibitor resistant subpopulations, providing a strategy to overcome drug resistance that might have relevance in broader therapeutic settings.

## Graphical Abstract

**Figure:**
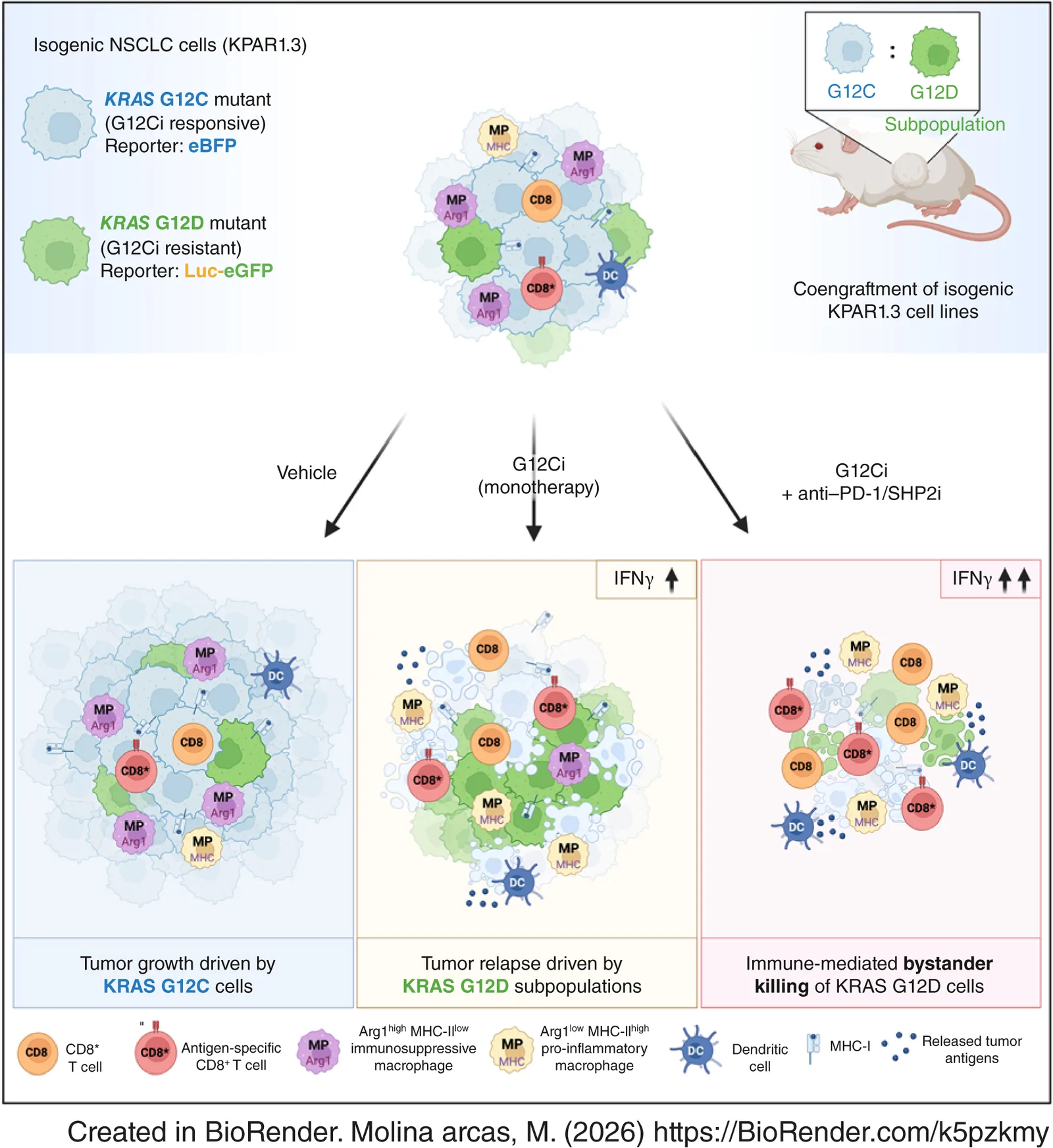

## Introduction

*KRAS* is the most frequently mutated oncogene in human cancer, with an incidence rate of approximately 90% in pancreatic adenocarcinoma, 50% in colorectal cancer, and 30% in lung adenocarcinoma (1). KRAS proteins cycle between a GDP-bound (off) state and a GTP-bound (on) state, the latter being able to interact with and activate downstream effector proteins such as RAF and PI3K (2). In mutant KRAS proteins, GTP hydrolysis is impaired, leading not only to promotion of aberrant growth and survival signaling but also to evasion of antitumor immunity (3, 4).

Recent advances in the development of inhibitors that directly target KRAS have opened new treatment opportunities for eligible patients with cancer (5, 6). Phase II clinical trials for the KRAS G12C (off) inhibitors adagrasib and sotorasib showed promising results, with most patients responding initially, leading to accelerated FDA approval (7, 8). However, most patients developed resistance within months and a subsequent phase III trial for sotorasib showed no overall survival advantage over docetaxel (9). Following on from the development of KRAS G12C (off) inhibitors, active-state RAS (on) G12C–selective inhibitors have also entered clinical trials (10). The most advanced of these are molecular glues that bind a ubiquitously expressed chaperone, cyclophilin A, forming a binary complex that selectively and covalently binds RAS G12C (GTP), preventing interaction with downstream effectors. Early clinical data show initial responses in most patients, including those who previously progressed on KRAS G12C (off) inhibitors (11). In parallel, many drugs targeting other mutant forms of KRAS and also unmutated RAS proteins are in advanced clinical development (4). Long-term data are not yet available, but resistance is also anticipated to be very likely to occur.

Resistance to KRAS inhibitors can develop through many different mechanisms. These include the presence of preexisting tumor subclones harboring alternative *KRAS* mutations that are intrinsically insensitive to KRAS G12C inhibition (12), acquisition of further mutations in oncogenic KRAS proteins or related signaling components (13, 14), genomic amplification or overexpression of pathway components, including KRAS itself (15, 16), or activation of alternative pathways, such as epithelial-to-mesenchymal transition (EMT) or YAP signaling (17, 18). These mechanisms likely emerge from subclonal events and are selected during therapy, although adaptive resistance due to signaling pathway rewiring can also occur (19). To overcome the rapid development of resistance, combination with other therapies is an attractive option (20).

Numerous preclinical studies with KRAS inhibitors have demonstrated not only tumor control and regression but also reversal of immune suppression (4, 21). KRAS inhibition restored antigen presentation and interferon (IFN) γ responses while reducing expression of immune checkpoint molecules such as PD-L1 and CD47 (22–25). In the tumor microenvironment (TME), antigen-presenting and CD8^+^ cytotoxic T-cell counts were increased (26, 27). On a systemic level, tumor control by KRAS inhibitors was superior in immunocompetent, compared with immune-compromised, mice and complete responder mice presented improved antitumor-directed immune memory (28, 29). Furthermore, inhibition of oncogenic KRAS signaling was found to sensitize responses toward immune checkpoint blockade (bioRxiv 2025.02.28.640711; refs. 23, 24).

Based on the preclinical findings, combination of KRAS inhibitors with immune-enhancing therapies, such as anti–PD-1/PD-L1 immunotherapy, has emerged as a promising clinical strategy currently under evaluation. When used as a single agent in lung cancer, immunotherapies lead to a subset of patients achieving durable responses, but the majority of patients display primary resistance to this treatment (30, 31). SHP2 inhibitors (SHP2i) also present an interesting therapeutic strategy, by vertically reinforcing inhibition of oncogenic KRAS signaling in cancer cells (16), while simultaneously inducing beneficial changes in other TME cell populations, such as reducing cytotoxic T-cell exhaustion, shifting macrophages to a less immunosuppressive polarized state, and normalizing tumor vasculature (32–34). Although first-generation KRAS inhibitors were limited by off-target toxicities that hindered their use in combination with these immunotherapies (35), newer generations with improved specificity may overcome these challenges and offer a therapeutic window for effective combination strategies.

Because rapid development of drug resistance is proving to be a key limitation for KRAS inhibitors, in this study we set out to explore novel methods for counteracting this phenomenon. As KRAS signaling promotes an immunosuppressive TME that is transiently reversed by KRAS inhibition, we hypothesized that KRAS inhibitor–induced killing of tumor cells could help prime an adaptive antitumor immune response. Immunogenic mechanisms of cell death may help promote such a targeted response (36, 37), together with reversal of the immunosuppressive makeup of the TME. Because most drug-resistant subpopulations of tumor cells would be expected to be very closely related to the drug-sensitive parental cells and share common antigens, any such immune priming could potentially act to promote destruction of the drug-resistant cells as well.

Using a mouse cancer model that mimics tumors containing a minor KRAS G12C inhibitor (G12Ci)–insensitive subpopulation, we show that although G12Ci as monotherapy conveyed a proliferative advantage to drug-resistant tumor subpopulations, combination therapies with an SHP2i or anti–PD-1 resulted in immune rejection of drug-resistant tumor subpopulations, complete responses, and enhanced immune memory, even though SHP2i or anti-PD-1 as monotherapy did not affect the growth of the drug-resistant tumors in isolation. Mechanistic studies revealed an increase in inflammatory-associated signatures in G12Ci-resistant cells and a profound, therapy-mediated remodeling in both innate and adaptive immune compartments in the TME.

This work establishes the paradigm that it is possible to induce immune-mediated bystander killing of drug-resistant subpopulations by appropriate combinations of oncogene-targeted inhibitors and immune-targeting agents. It further provides a platform to explore this novel approach to countering drug resistance in cancer therapy, including gaining further mechanistic insights and optimal strategies for potentiating this effect.

## Materials and Methods

### Cell culture conditions and treatments

KPAR1.3 G12D and KPAR1.3 G12C cell lines, as well as *Ifngr*-knockout (KO) and *Emv2*-KO KPAR1.3 G12D cells, were generated as previously described (28, 38). All KPAR1.3 cell lines, including derivatives established for this study, were maintained in DMEM (Gibco, 41966052) supplemented with 10% fetal calf serum, 4 mmol/L glutamine, and 100 U/mL penicillin/streptomycin and were passaged using trypsin (Gibco, 25200072). Cell lines were tested regularly for *Mycoplasma* by the Cell Science facility at The Francis Crick Institute.

For the different *in vitro* experiments, cells were plated at appropriate densities and treated 24 hours after seeding. RMC-4998 was provided by Revolution Medicines under a sponsored research agreement. RMC-4550 was provided by Revolution Medicines under a former collaboration agreement with Sanofi. Adagrasib (MRTX849) was obtained from MedChemExpress and trametinib (GSK1120212), pictilisib (GDC-0941), and mitoxantrone from Selleck Chemicals. Recombinant mouse IFNγ was obtained from BioLegend.

### Generating reporter-traced KPAR1.3 cell lines

The lentiviral plasmids pLARRY-eGFP (#140025; RRID: Addgene_140025) and pFUGW-FerH-ffLuc2-eGFP (#71393; RRID: Addgene_71393) were obtained from Addgene. The nucleotide sequence of pLARRY-eGFP was altered to encode enhanced blue fluorescent protein (eBFP) by substitution of two individual nucleotides via site-directed mutagenesis using the In-Fusion Snap Assembly Master Mix (TaKaRa, 638947) and the primers Tyr67 to His (Fwd: ccc​tga​ccc​acg​gcg​tgc​agt​gct​tca​g; Rev: cgc​cgt​ggg​tca​ggg​tgg​tca​cga​gg) and Tyr146 to Phe (Fwd: gta​caa​ctt​caa​cag​cca​caa​cgt​cta​tat​cat​g; Rev: ctg​ttg​aag​ttg​tac​tcc​agc​ttg​tgc​cc). PCR conditions for rolling cycle amplification were performed following the manufacturer’s instructions. Edited plasmids were transformed into *Stbl2 Escherichia coli* using a standard heat-shock protocol for selection and subsequent plasmid purification.

Reporter constructs were introduced into KPAR1.3 cells via lentiviral transduction. Lentivirus were generated by cotransfecting HEK293T cells (RRID: CVCL_0063) with reporter plasmids (pLARRY-eBFP and pFUGW-FerH-ffLuc2-eGFP) and packaging plasmids (pCMV-dR8.2; RRID: Addgene_8455 and pCMV-VSV-G; RRID: Addgene_8454) using the jetPRIME Transfection Kit (Polyplus, 101000015). After 48 hours, virus-containing supernatant was collected, filtered (0.45 μm), and used for spinning infection of KPAR1.3 cells (600 rpm, 90 minutes, room temperature). Fluorescent reporter-expressing cells were single-cell cloned by FACS into 96-well plates.

### Characterizing reporter-traced KPAR1.3 subclones *in vitro*

For growth-rate, viability, and luciferase activity assays, cells were plated into cell culture–treated 96-well plates with 1,500 cells in 100 μL of the standard cell culture media, described above. For viability assays, inhibitors were added in serial dilution, as indicated in respective experiments. Viability was measured by adding 5 μL CellTiter-Blue (Promega, G8081), incubating for 90 minutes at 37°C, and detecting fluorescence with an EnVision 2105 multimode plate reader (PerkinElmer). For growth-rate assays, confluency was monitored using S3 Incucyte (Sartorius) at 20× magnification. Wells were imaged every 6 hours for up to 5 days or until confluency. Confluency over time was quantified using S3 Live-Cell Imaging and Analysis software (Sartorius). *In vitro* luciferase activity was assessed using the Pierce Firefly Luciferase Glow Assay Kit following the manufacturer’s instructions (Thermo Fisher Scientific, 16176). Bioluminescence was measured with an EnSight multimode plate reader (PerkinElmer).

### Western blotting

Cells were lysed in cell lysis buffer (Cell Signaling Technology, 9803) supplemented with cOmplete Mini protease inhibitor cocktail and PhosSTOP phosphatase inhibitors (Roche). Lysates were purified via centrifugation at 15,000 rpm for 15 minutes at 4°C, and the protein concentration was measured for normalization using the detergent compatible Protein Assay Kit (Bio-Rad, 5000111). Twenty micrograms of protein was then prepared with 4× NuPAGE LDS Sample Buffer (Invitrogen, NP0007), separated on NuPAGE 4% to 12% Bis–Tris gels (Invitrogen), and transferred to polyvinylidene difluoride (PVDF) or nitrocellulose membranes (Merck Millipore) for chemiluminescence or fluorescence detection, respectively. The membranes were incubated with the indicated primary antibodies (Supplementary Table S1). Bound primary antibodies were incubated with the appropriate horseradish peroxidase (HRP)–conjugated secondary antibodies, and protein expression was detected via chemiluminescence using Immobilon Western HRP substrates (Merck). Alternatively, the membranes were incubated with secondary antibodies compatible with infrared detection at 700 and 800 nm and scanned using the Odyssey Infrared Imaging System (Odyssey, LICORbio).

### Quantitative reverse-transcription PCR

RNA was extracted using the RNeasy Mini Kit (Qiagen) following the manufacturer’s instructions. cDNA was generated using the Maxima First Strand cDNA Synthesis Kit (Thermo Fisher Scientific). qPCR was performed using Fast SYBR Green reagents (Applied Biosystems). Gene expression changes relative to the housekeeping genes were calculated using the ΔΔCT method. The list of primers used is shown in Supplementary Table S2.

### HMGB1 immunoassay

Twenty thousand KPAR1.3 KRAS G12C cells were plated in 100 μL complete DMEM in 96-well plates and treated 24 hours later. After 48-hour treatment, supernatants were harvested and HMGB1 release was measured using Lumit HMGB1 Immunoassay (Promega, W6112) following the manufacturer’s instructions.

### *In vivo* techniques

All *in vivo* experiments were approved by the Animal Welfare and Ethical Review Board of The Francis Crick Institute and conducted according to local guidelines and UK Home Office regulations under the Animals Scientific Procedures Act 1986. Transplantation experiments were performed using either C57BL/6J mice (RRID: IMSR_JAX:000664) or albino “glowing head” (GH) C57BL/6J mice [C57BL/6-Tyr^c-Brd^ Tg (Gh1-luc/EGFP) D8Mrln/J; ref. 39]. Alternatively, for experiments in immune-compromised mice GH mice were crossed with *Rag1*^−/−^ mice (RRID: IMSR_JAX:002216). When possible, cohorts included both male and female mice with balanced distribution across treatment groups. However, this was occasionally limited by animal availability.

For subcutaneous engraftment studies, 7- to 12-week-old mice were engrafted with a cell suspension of 150,000 KPAR1.3 cells, which were prepared in 100 μL of 50% PBS and 50% Geltrex (Gibco, A1413202). Tumor growth was measured twice weekly via caliper, and the volume was calculated as (length × width^2^)/2. Mice were culled via cervical dislocation when tumors reached a diameter of 1.2 to 1.4 cm or if any other humane endpoint was reached. Mice that rejected their primary subcutaneous tumor and remained tumor free (cutoff: 9 mm^3^, to account for residual stromal tissue and similar lesions) for ∼4 weeks after treatment were classified as complete responders (CR). These mice were rechallenged with 150,000 luciferase–enhanced green fluorescent protein (Luc-eGFP) G12Ci-resistant cells injected into the opposite flank, unless otherwise stated.

For *in vivo* imaging of Luc-eGFP–expressing KPAR1.3 KRAS G12D cells, mice were injected intraperitoneally with 100 μL 30 μg/mL XenoLight D-Luciferin (PerkinElmer, 122799) dissolved in PBS and anesthetized via inhalation of 2% to 2.5% isoflurane vaporized in O_2_. Bioluminescence was imaged in mice using the IVIS SpectrumCT scanner (PerkinElmer; RRID: SCR_014247), and data were analyzed using Living Image software (versions 4.4–4.5, PerkinElmer).

The inhibitors adagrasib (MedChemExpress, 50 mg/kg), RMC-4998 (Revolution Medicines, 100 mg/kg), and RMC-4550 (Revolution Medicines, 30 mg/kg) were prepared in a 50 mmol/L sodium citrate buffer (pH 5) containing 10% w/v Captisol (Ligand). Drugs were administered via oral gavage, once daily for a maximum of 6 days a week. Anti–PD-1 (Bio X Cell; RRID: AB_10949053; clone RMP1-14, 10 mg/kg) was prepared in PBS and administered twice weekly via intraperitoneal injection. Combinations and durations of treatments are indicated in respective experiments.

### Flow cytometric techniques

Tumors were first minced with a scalpel, followed by enzymatic dissociation using mouse Tumor Dissociation Kit (Miltenyi Biotec, 130-096-730). The resulting cell suspension was filtered through a 70-μm MACS SmartStrainer (Miltenyi Biotec, 130-110-916). Red blood cells (RBC) were lysed with 5 mL ACK lysing buffer (Gibco, A1049201), followed by a washing step with FACS buffer, prepared as PBS containing 2% FBS and 1 mmol/L ethylenediaminetetraacetic (EDTA). For quantification of tumor cell subpopulations via their fluorescent protein expression, live-cell suspensions were stained with viability dye DRAQ7 (1:100) and analyzed on BD LSRFortessa X-20 cell analyzers (BD Biosciences) using corresponding BD FACSDiva (RRID: SCR_001456) and BD FlowJo analysis software (version 10.8.1–10.8.3; RRID: SCR_008520).

For immunophenotyping via spectral flow cytometry, additional single-cell suspensions from spleens and tumor-draining lymph nodes (tdLN) were obtained by directly pushing respective tissues through a 70-μm MACS SmartStrainer, followed by ACK lysis for spleen suspensions. All samples were then transferred to conical 96-well plates and normalized to ∼1.5 × 10^6^ cells per well. Before staining, cell suspensions were blocked with CD16/CD32 Mouse BD Fc Block (BD Biosciences, 553141; RRID: AB_394657) for 15 minutes. A staining panel master mix for extracellular staining was prepared (antibodies and viability dye listed in Supplementary Table S3), and cells were stained for 20 minutes at room temperature. Single staining controls were prepared using UltraComp eBeads (Invitrogen, 01-2222-42). Cells were then fixed/permeabilized using Foxp3 fixation/permeabilization buffer (Invitrogen, 00-5523-00) for 30 minutes at 4°C and washed with the buffer provided in the same kit. Samples were then stained with the intracellular antibody master mix (Supplementary Table S3) for 20 minutes at room temperature, washed, and resuspended in FACS buffer. Immunophenotyping was performed on the Cytek Aurora spectral flow cytometer (RRID: SCR_027060), with spectral unmixing using Cytek SpectroFlo software (RRID: SCR_025494) and further analysis in FlowJo (versions 10.8.1–10.10.1/RRID: SCR_008520). Gating strategy is shown in Supplementary Fig. S1.

For the analysis of Emv2-specific T cells, initial sample processing was performed as described above. Once samples were transferred to a 96-well plate, cells were first incubated in PBS containing CD16/CD32 Mouse BD Fc Block (BD Biosciences, 553141) and Zombie NIR viability dye for 15 minutes at room temperature. Next, samples were stained with 2× T-select H-2Kb MuLV p15E Tetramer-KSPWFTTL (MBL, MBL-TS-M507-1) for 40 minutes at 4°C. Subsequently, extracellular master mix, prepared with the antibodies listed in Supplementary Table S4 and diluted in Foxp3 fixation/permeabilization buffer (Invitrogen, 00-5523-00), was added, and samples were incubated for 30 minutes at 4°C. Fixation, intracellular staining, and sample immunophenotyping on a Cytek Aurora spectral flow cytometer were performed as described above. Gating strategy is shown in Supplementary Fig. S2.

### Quantification of tumor-binding antibodies

To assess whether serum from tumor-bearing mice contained tumor cell–opsonizing antibodies, serum samples were collected from mice harboring KPAR1.3 KRAS G12C tumors, from mice with KPAR1.3 KRAS-G12D *Emv2-*KO tumors, and from naïve mice without tumors. To test for the presence of tumor cell–specific antibodies, 3 × 10^5^ KPAR1.3 cells were plated per well in a 96-well plate for each isogenic version of the cell line: (i) KRAS G12C, (ii) KRAS G12D, and (iii) KRAS G12D *Emv2-*KO. Samples were incubated with 3 μL of mouse serum and diluted in 100 μL FACS buffer per well for 1 hour at 4°C. Cells were then stained with FITC-conjugated anti–mouse IgG antibody (BioLegend, 406001; RRID: AB_315029) for 30 minutes at 4°C. DAPI was added at working concentrations to exclude dead cells. Samples were acquired on a BD LSRFortessa X-20 cell analyzer (BD Biosciences), and data were analyzed using FlowJo (v10; RRID: SCR_008520). The median fluorescence intensity (MFI) of FITC was used as a measure of antibody binding. For each condition, FITC MFI values were normalized to those obtained from serum of naïve mice.

### Bulk RNA sequencing

To ensure sufficient material for downstream analysis, especially in the vehicle and SHP2i conditions, a higher proportion (7.5%) of KRAS G12D cells was coengrafted for the experiment combining G12Ci plus SHP2i. When all groups contained G12Ci, the ratio of engrafted KRAS G12C cells was reduced to 0.5%. After treatment, subcutaneous tumors were extracted, dissociated into single-cell suspensions, and RBC lysed using ACK lysis buffer (Gibco, A1049201).

To eliminate additional autofluorescent events in the eBFP+ sort gate, CD45^+^ immune cells were removed previously via magnetic pulldown, using mouse CD45 MicroBeads (Miltenyi Biotec, 130-052-301). Cancer cells were then flow cytometrically sorted according to their expressed fluorescent proteins, and RNA was purified using the RNeasy Micro Kit (Qiagen, 74004). Alternatively, for *in vitro* characterization of the KPAR.E2 cell line, parental KPAR1.3 G12C and KPAR.E2 cells were treated for 24 hours with DMSO or 100 nmol/L adagrasib, and RNA was extracted using the RNeasy Mini Kit (Qiagen) following the manufacturer’s instructions. The Genomics Science Technology Platform at The Francis Crick Institute prepared reverse-stranded sequencing libraries from 50 ng of RNA using either NEBNext Ultra II Directional PolyA mRNA (New England Biolabs) or the Watchmaker mRNA Library Prep Kit (modules K0105 and K0078), following the manufacturer’s protocols. Paired-end sequencing (100 bp) was performed on an Illumina NovaSeq X platform, with a minimum depth of 25 million reads per sample.

Sequencing results were first preprocessed utilizing the nf-core pipeline (v3.14.0), selecting the *star_rsem* aligner (RRID: SCR_004463/RRID: SCR_000262) and the mouse reference genome version Mus_musculus.GRCm38.95 for alignment of sequenced reads. Data analysis was conducted using the DESeq2 pipeline (RRID: SCR_015687), and the hallmark gene set collection from the MSigDB website (https://www.gsea-msigdb.org/gsea/msigdb/; RRID: SCR_016863) was used for gene set enrichment analysis (GSEA).

### Multiplex immunohistochemistry

Stainings of formalin-fixed, paraffin-embedded tissue sections were performed by the Experimental Histopathology core facility at The Francis Crick Institute. Experiment was done twice to obtain two independent biological replicates. Tumors were fixed in 10% neutral buffered formalin for 48 hours, stored in 70% EtOH, and embedded in paraffin wax using a Tissue-Tek VIP 6 AI processor (Sakura). Sections (3 μm) were cut and mounted on glass slides, which were baked at 60°C for 1 hour to adhere the tissue and melt the wax. Slides were then transferred to a Leica Bond RX platform (Leica, 3498240), in which a 0.1% BSA solution for protein blocking and a 3% hydrogen peroxide solution for inhibiting endogenous peroxidases were applied to prepare the slides for staining. Antigens were then sequentially stained using HRP-conjugated antibodies and various Opal dyes (Akoya). The antibodies targeting the following epitopes were used: eGFP/eBFP, clone A-6455, Thermo Fisher Scientific, RRID: AB_221570; CD4, clone EPR19514, Abcam, RRID: AB_2686917; cleaved caspase 3 (Casp3), clone D3E9, Cell Signaling Technology, RRID: AB_2341188; CD8a, clone EPR21769, Abcam, RRID: AB_2890649; luciferase, NB100-1677, Novus Biologicals, RRID: AB_10000585; and Ki67, ab15580, Abcam, RRID: AB_443209. Between each antigen staining, slides underwent antibody stripping and antigen retrieval by heating at 95°C for 20 minutes with epitope retrieval solutions (Leica, AR9961 and AR9640). Nuclei were then stained with DAPI (Thermo Fisher Scientific, 62248) and coverslips mounted using ProLong Gold Antifade reagent (Invitrogen, P36934). Finally, slides were scanned on a PhenoImager HT (Akoya, VP2143N2122) in MOTiF scanning mode using Vectra Polaris software (version 1.0.13), and unmixing of fluorescent spectra was performed via appurtenant InForm software (version 2.6.0).

Tumor areas for analysis were first annotated using QuPath (version 0.4.3; RRID: SCR_018257), with the very outer perimeter excluded to minimize detached edges and staining edge effects. Larger necrotic regions were identified based on Casp3 expression, in consultation with experimental histopathology, and were also excluded from analysis. Additional technical artifacts such as wrinkles, rips, or tissue folds were similarly annotated and excluded from downstream processing. Because stain intensity varied across sections, a mean background subtraction to balance uneven illumination was performed. A Gaussian blur was also performed to smooth out features and improve downstream analysis. Nuclei were segmented with StarDist (arXiv:1908.03636, arXiv:2203.02284; ref. 40), a U-Net–based approach trained and benchmarked on our dataset. In parallel, semantic masks for CD4, CD8, GFP/BFP, luciferase, Ki67, and Casp3 were generated using a trained random-forest classifier (https://doi.org/10.5281/zenodo.3555620). Cell types were assigned based on the intersection between nuclear masks with the corresponding marker masks (CD4, CD8, GFP/BFP, and luciferase). Conflicts between markers were resolved using prespecified thresholds. Cytoplasm was delineated without a membrane marker under three constraints. (i) Cytoplasm surrounds its nucleus and is spatially bounded; we created a mask based on the position of each positive nucleus and expanded it into their corresponding, overlapping marker masks. (ii) Cytoplasm from different cells does not overlap; overlaps were resolved by Euclidean Voronoi reassignment of pixels to the nearest cell center of mass, with nuclei reinforced as true positives. (iii) Cytoplasm is contiguous; after Voronoi assignment, fragmented pixel islands were reassigned to the adjacent cell with the greatest connected support. Isolated pixels with no neighboring cells were discarded.

### Statistical analysis

Data were compared using the pair-end Student *t* test or analysis of variance (ANOVA) if more than two experimental groups were analyzed. The test used for each experiment is indicated in the figure legends. Significance was determined at *P* < 0.05 (*, *P* < 0.05; **, *P* < 0.01; ***, *P* < 0.001; ****, *P* < 0.0001).

### Illustrations

Graphical schematics were created using BioRender.com.

## Results

### A tumor model to mimic development of G12Ci resistance as a subclonal event in solid tumors

To establish a model system to study the emergence of resistance to KRAS G12C inhibition, we used two isogenic variants of the KPAR1.3 lung cancer cell line (28), harboring either *KRAS* G12C or *KRAS* G12D mutations. The *KRAS* G12D variant is insensitive to G12Cis and was used to model preexisting tumor subclones that are not eliminated by G12Ci treatment, reflecting clinically relevant mechanisms of drug resistance (13, 14). Therefore, coengraftment of these two isogenic cell lines, which form immune-hot tumors, enabled modeling the presence of minor drug-insensitive subpopulations within an otherwise drug-sensitive tumor.

To enable tracking of the coengrafted KPAR1.3 *KRAS* G12C– and *KRAS* G12D–mutant cancer cells *in vitro* and *in vivo*, we first transduced the isogenic cell lines with reporter constructs. To avoid artifactual immune responses against reporter proteins, we employed the GH mouse model, which expresses a Luc-eGFP fusion construct in the anterior pituitary gland (39). The KRAS G12D cell line was transduced with Luc-eGFP construct, enabling tracking of the KRAS G12D subpopulation *in vivo* via bioluminescence imaging. In parallel, the KRAS G12C cells were transduced with eBFP, which differs from eGFP by only two substitutions (Supplementary Fig. S3A). The additional peptides derived from eBFP exhibited very weak MHC-l–binding properties (Supplementary Table S5), suggesting that eBFP is unlikely to be recognized as foreign in eGFP-tolerant mice.

**Figure 1.**
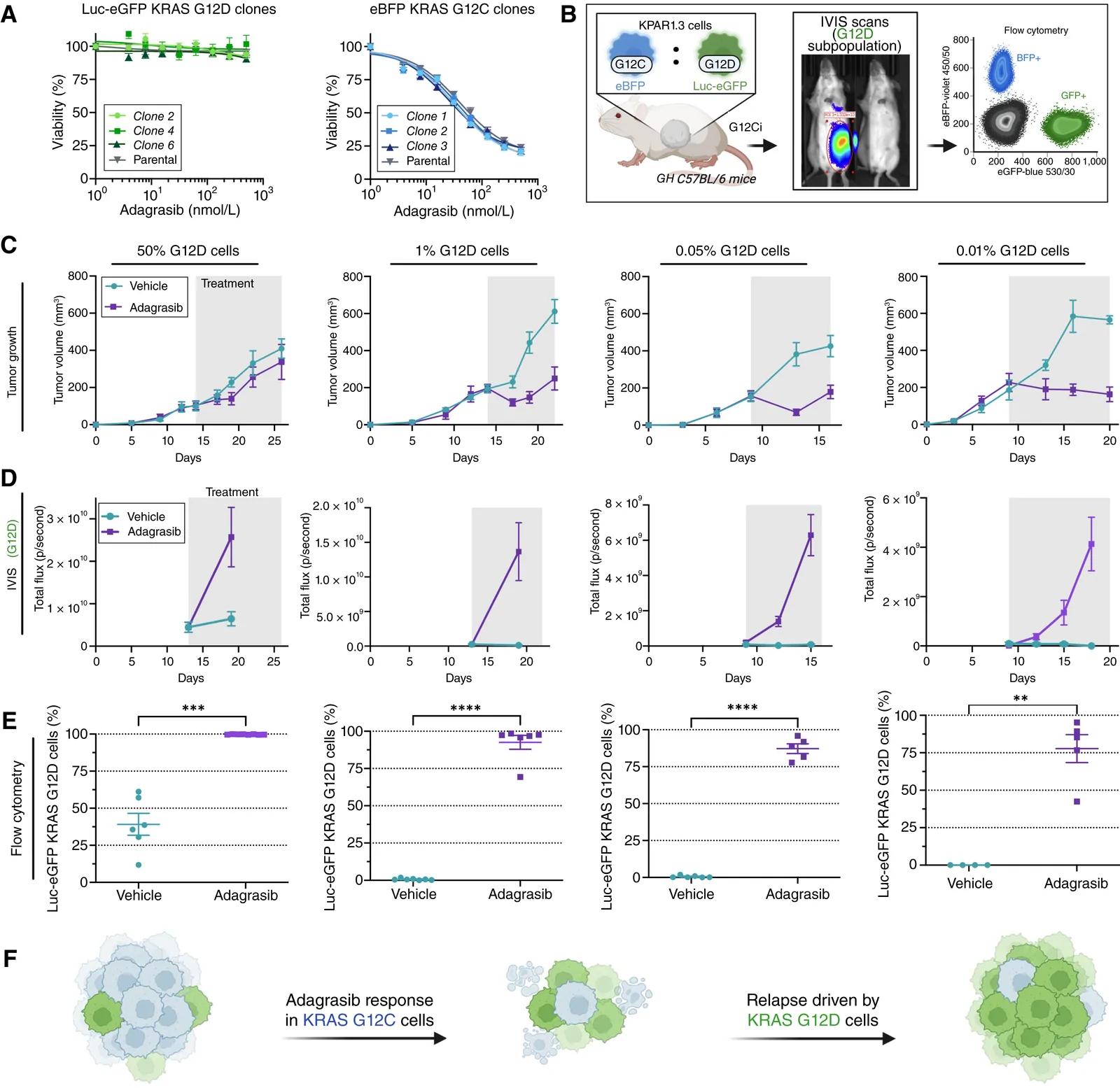
G12Ci as monotherapy promotes the outgrowth of drug-resistant subpopulations. **A,** Viability assays of Luc-eGFP KRAS G12D or eBFP KRAS G12C KPAR1.3 subclones compared with the parental counterparts. Cells were treated with adagrasib for 72 hours. Mean ± SEM of three biological replicates (*n*) with three technical replicates. **B,** Experimental setup for **C–E**. Subcutaneous tumors were engrafted with different ratios of eBFP KRAS G12C and Luc-eGFP KRAS G12D cells, as indicated. Mice were treated daily with the G12Ci adagrasib (50 mg/kg; gray area). *n* = 6–8 mice per group. **C,** Tumor growth over time for indicated ratios of KRAS G12C:KRAS G12D cells. **D,** Bioluminescence scans over time, detecting signal emitted by luciferase-expressing KRAS G12D cells. **E,** Flow cytometric analysis of Luc-eGFP KRAS G12D cells of all live cancer cells. All graphs display mean ± SEM; pair-end Student *t* test with Welch correction. **F,** Schematic of subpopulation dynamics. ***, *P* < 0.001; ****, *P* < 0.0001.

The transduced cell lines were then subcloned and characterized *in vitro* to confirm retention of parental growth rates and comparable growth between KRAS G12C and KRAS G12D cells (Supplementary Fig. S3B). Luciferase activity was demonstrated for the KRAS G12D clones (Supplementary Fig. S3C). As expected, only the KRAS G12C clones were sensitive to the KRAS G12C (off) inhibitor adagrasib (Fig. 1A), while similar responses to MEK inhibition were observed (Supplementary Fig. S3D). Finally, we assessed how population dynamics evolved during continuous adagrasib treatment in mixed KRAS G12C and KRAS G12D cocultures *in vitro* (Supplementary Fig. S3E).

To confirm comparable proliferation rates *in vivo*, reporter-traced KRAS G12C and KRAS G12D cell lines were coengrafted subcutaneously at a 50/50 ratio in GH mice. Cancer cell fractions from untreated tumors were determined via flow cytometry and spatial distribution using immunohistochemistry (Supplementary Fig. S4). In these stainings, the GFP antibodies recognize both cancer cell populations as they cross-react with the closely related BFP protein, whereas the luciferase antibodies recognize only the KRAS G12D cells.

To model the early onset of treatment resistance in a preclinical setting, we next established a murine model system harboring KPAR1.3 KRAS G12C–derived tumors coengrafted with a subpopulation of G12Ci-insensitive cells (*KRAS* G12D mutant). Given prior evidence that G12Cis can at least partially reverse immune suppression in responsive tumors and induce complete tumor regressions in some mice (24), we investigated whether G12Ci as monotherapy could elicit antitumor immunity capable of targeting both sensitive and resistant clones through recognition of shared tumor antigens. To test this, *in vivo* experiments with four different engraftment ratios of eBFP KRAS G12C and Luc-eGFP KRAS G12D cells in a subcutaneous setting were performed (Fig. 1B). Responses in vehicle- or adagrasib-treated mice were assessed via measurement of tumor growth (Fig. 1C), and the abundance of KRAS G12D cells over time was measured via bioluminescence scans (Fig. 1D). Additionally, the fraction of KRAS G12D cells of all live tumor cells at the end of treatment was determined via flow cytometry (Fig. 1E). Even when engrafting very few G12Ci-resistant cells (equivalent to 15 cells of 150,000 engrafted cells), the resistant subpopulation gained a strong proliferative advantage in adagrasib-treated tumors, with the majority of tumors being mostly comprised of KRAS G12D cells within 2 weeks of treatment. From this series of experiments, we conclude that treatment with adagrasib promotes the outgrowth of G12Ci-resistant tumor subpopulations and that any potential enhanced immune response against shared tumor antigens is not sufficient to generate complete responses, even when only very small numbers of drug-resistant cells are present (Fig. 1F).

### Combination therapies enhance treatment responses and promote elimination of G12Ci-resistant subpopulations

We next wanted to assess whether combining KRAS G12C inhibition with other therapies, known to bolster antitumor immunity, may promote immune attack on G12Ci-resistant tumor subpopulations. We selected an immune checkpoint blockade antibody targeting PD-1 (30) and an SHP2i (RMC-4550), which acts through pleiotropic mechanisms both in cancer cells and immune cells (32–34).

*In vitro* experiments showed that cell death induced by G12Ci was associated with the release of HMGB1, a known marker of immunogenic cell death (36, 37), at levels comparable with the well-stablished immunogenic cell death inducer mitoxantrone (Supplementary Fig. S5A). Therefore, the death of G12Ci-sensitive cells could potentially prime an immune response that would target shared tumor antigens on both populations, providing a mechanistic basis for synergy with immune-enhancing therapies.

**Figure 2.**
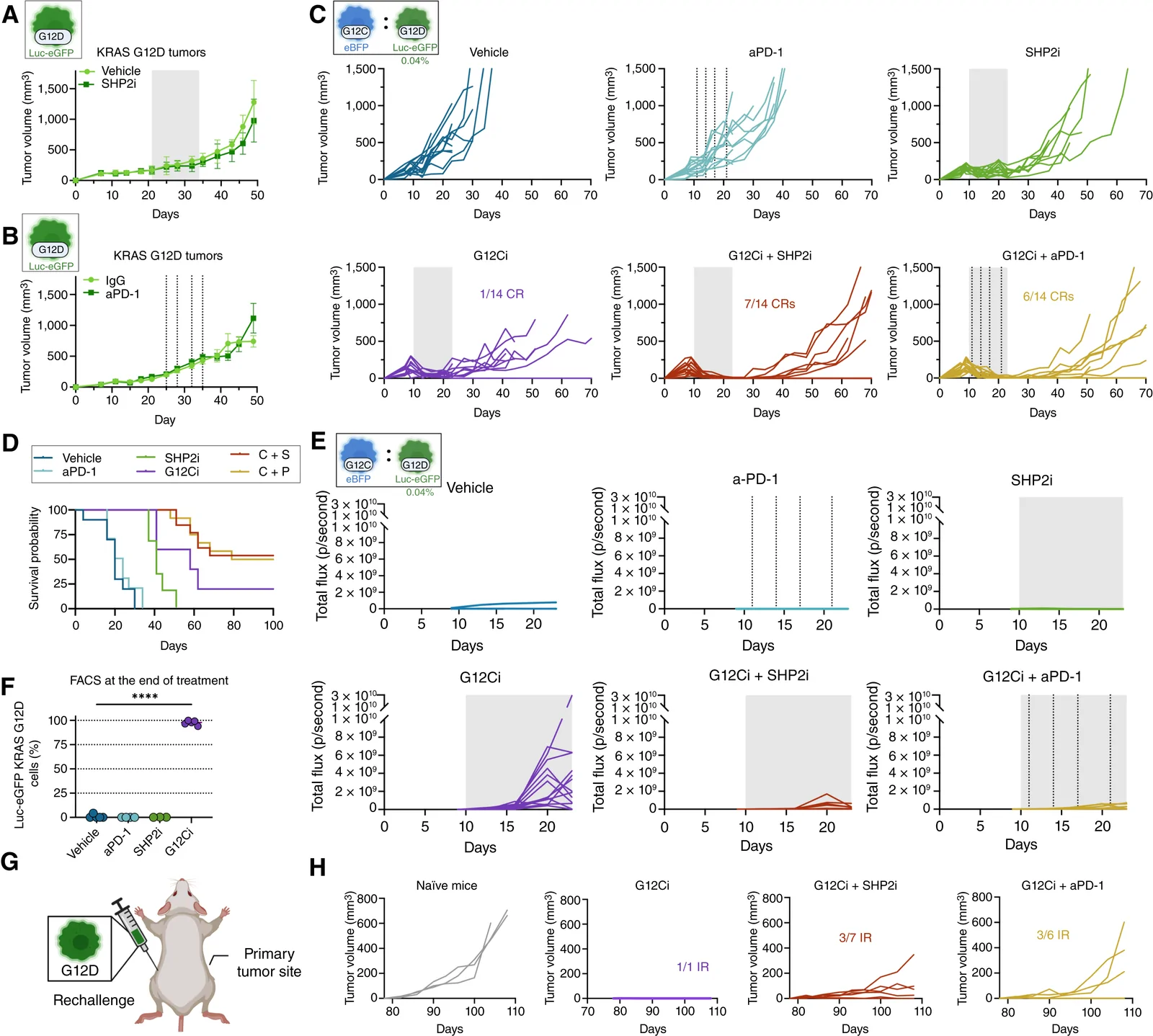
Combination therapies promote elimination of resistant subclones. **A** and **B,** Luc-eGFP KRAS G12D KPAR1.3 subcutaneous tumors treated with vehicle (*n* = 7) or RMC-4550 (SHP2i; 30 mg/kg; *n* = 8)(**A**); isotype control (10 mg/kg, IgG, *n* = 6) or anti–PD-1 (aPD-1; 10 mg/kg; *n* = 7) (**B**). Doses indicated with gray area (SHP2i) or vertical dotted lines (anti–PD-1). Mean ± SEM. **C–F,** Mixed subcutaneous tumors (BFP KRAS G12C cells plus 0.04% Luc-eGFP KRAS G12D cells) were treated for 2 weeks with RAS G12C (on) inhibitor RMC-4998 (G12Ci; 100 mg/kg) ± SHP2i RMC-4550 (30 mg/kg) or anti–PD-1 (10 mg/kg). Tumor volume over time of individual tumors. Complete responder (CR) mice are indicated in respective plots (**C**). Survival probability across different treatment groups. Cutoff value: 500 mm^3^. C, G12Ci; P, aPD-1; S, SHP2i (**D**). Bioluminescence scans, indicating the relative abundance of Luc-eGFP KRAS G12D cells, measured in total flux, photons per second (p/second) (**E**). Representative tumors were isolated from vehicle and single-therapy treatments, and the fraction of Luc-eGFP KRAS G12D cells within all cancer cells was determined via flow cytometry. Each dot represents one tumor; mean ± SEM; one-way ANOVA test comparing each of the treatment groups with the vehicle (**F**). **G,** Rechallenge experiment: CR mice from **C** were injected in the opposite flank with Luc-eGFP KRAS G12D cells. **H,** Tumor growth over time of rechallenged tumors in naïve mice or CRs. Mice that immune-rejected (IR) the re-injected cancer cells are indicated. Graph titles indicate the treatment that the primary tumor received. ****, *P* < 0.0001.

First, we evaluated single-agent responses *in vivo* in immunocompetent mice subcutaneously engrafted with either the KRAS G12C or KRAS G12D cell line. In these studies, we used the active-state RAS (on) G12C–selective inhibitor, RMC-4998, a tool compound representative of the investigational drug candidate elironrasib (henceforth referred to as G12Ci; ref. 10). As expected, KRAS G12C–derived tumors were responsive to G12Ci treatment, with three of eight complete responders (CR) after 2 weeks of treatment followed by drug withdrawal (Supplementary Fig. S5B). KRAS G12C tumors also showed partial regression following SHP2 inhibition (Supplementary Fig. S5C), although no complete responders were observed, whereas anti–PD-1 treatment resulted in only modest tumor control (Supplementary Fig. S5D). Interestingly, KRAS G12D tumors did not show a detectable response to SHP2i (Fig. 2A). This is consistent with their lower *in vitro* sensitivity compared with KRAS G12C cells, particularly under reduced serum conditions (Supplementary Fig. S5E), and with previous studies showing that different *KRAS* mutations exhibit differential responses to SHP2 inhibition, probably due to biochemical differences that influence their dependence on upstream signaling (41, 42). KRAS G12D tumors also did not show a detectable response to anti–PD-1 treatment (Fig. 2B), demonstrating that these tumors are resistant to all three lines of therapy used here when administered as monotherapies.

Next, we assessed whether combinations could elicit durable responses in mice engrafted with KRAS G12C tumors containing a small subpopulation (0.04%) of G12Ci-resistant cells. In this mixed tumor setting, mice receiving G12Ci as monotherapy showed tumor regression during the treatment interval, with one mouse achieving a CR (Fig. 2C), whereas combining G12Ci with either SHP2i or anti–PD-1 led to complete and durable responses in approximately half of the mice. These responses were achieved despite the presence of KRAS G12D subpopulations that did not visibly respond to any of the administered treatments when growing as pure tumors. Synergistic effects of the combinations were also evident when visualizing survival probabilities (Fig. 2D).

Bioluminescence scans revealed further mechanistic insights into the dynamics of the tumor cell fractions, showing that the KRAS G12D subpopulation increased substantially during G12Ci treatment as monotherapy, whereas combination treatments demonstrated enhanced control of the G12Ci-resistant tumor subpopulation (Fig. 2E). Bioluminescence signal at the end of treatment correlated with the generation of complete responses, with most mice achieving CRs showing no clear outgrowth of the KRAS G12D subpopulation (Supplementary Fig. S5F).

Flow cytometric quantification of the KRAS G12D fraction at the end of treatment confirmed that the G12Ci promoted the expansion of the resistant subpopulation (Fig. 2F). Tumors from combination-treated mice could not be sampled at this timepoint, as they had regressed too much to obtain sufficient material. Additionally, to assess the cancer cell fractions of relapsed tumors in non-CRs, tumor samples were also analyzed at endpoint. This revealed a high degree of variability; whereas some tumors contained a mix of both tumor cell populations, others, particularly in the combination groups, were mostly comprised of either KRAS G12C or KRAS G12D cells (Supplementary Fig. S5G). This suggested that KRAS G12C cells can persist during treatment and, in some cases, reestablish proliferative capacity and contribute to the tumor mass in relapsed tumors.

To test if combination treatments further prime an adaptive immune response that enabled the recognition of shared epitopes between the isogenic cancer cell populations and contributed to the immune-mediated elimination of the drug-resistant subpopulations, CR mice were rechallenged with 150,000 KRAS G12D cells in the opposite flank (Fig. 2G). Compared with naïve mice, tumor-experienced mice either completely rejected the reinjected cells or showed delayed tumor growth, indicating the involvement of an adaptive immune response and immune memory (Fig. 2H). Similarly, immune rejection was observed when KRAS G12D cells were injected into mice that completely rejected KRAS G12C–only tumors (Supplementary Fig. S5H), consistent with the presence of antitumor immune memory capable of recognizing shared antigens.

We explored whether treatment responses in our mixed model could be further enhanced by using the triple-therapy combination. Although fewer CRs were observed in the dual-combination groups compared with the previous experiment, 12 of 14 mice in the triple-combination group were CRs, indicating additional synergism (Supplementary Fig. S6A). This correlated with the bioluminescence scans showing improved control of the G12Ci-resistant subpopulation at the end of the treatment (Supplementary Fig. S6B). CR mice were rechallenged by injecting KRAS G12D cells in the opposite flank, and results again supported the presence of antitumor-directed immune memory against shared antigens (Supplementary Fig. S6C). We speculated that the observed increase in CRs in triple combination–treated mice could reflect additional synergy between SHP2i and anti–PD-1. Indeed, SHP2i plus anti–PD-1 achieved tumor control in GH mice harboring either KRAS G12C– or KRAS G12D–derived tumors (Supplementary Fig. S6D), although no CRs were observed in either group.

Taken together, these data indicate that combining G12Ci with immune-enhancing therapies can generate complete responses in the presence of G12Ci-resistant tumor subpopulations, likely by promoting an adaptive immune response against tumor antigens shared between drug-sensitive and drug-insensitive cell populations.

### G12Ci induces early formation of proliferative clusters of KRAS G12D cells and extensive T-cell infiltration

**Figure 3.**
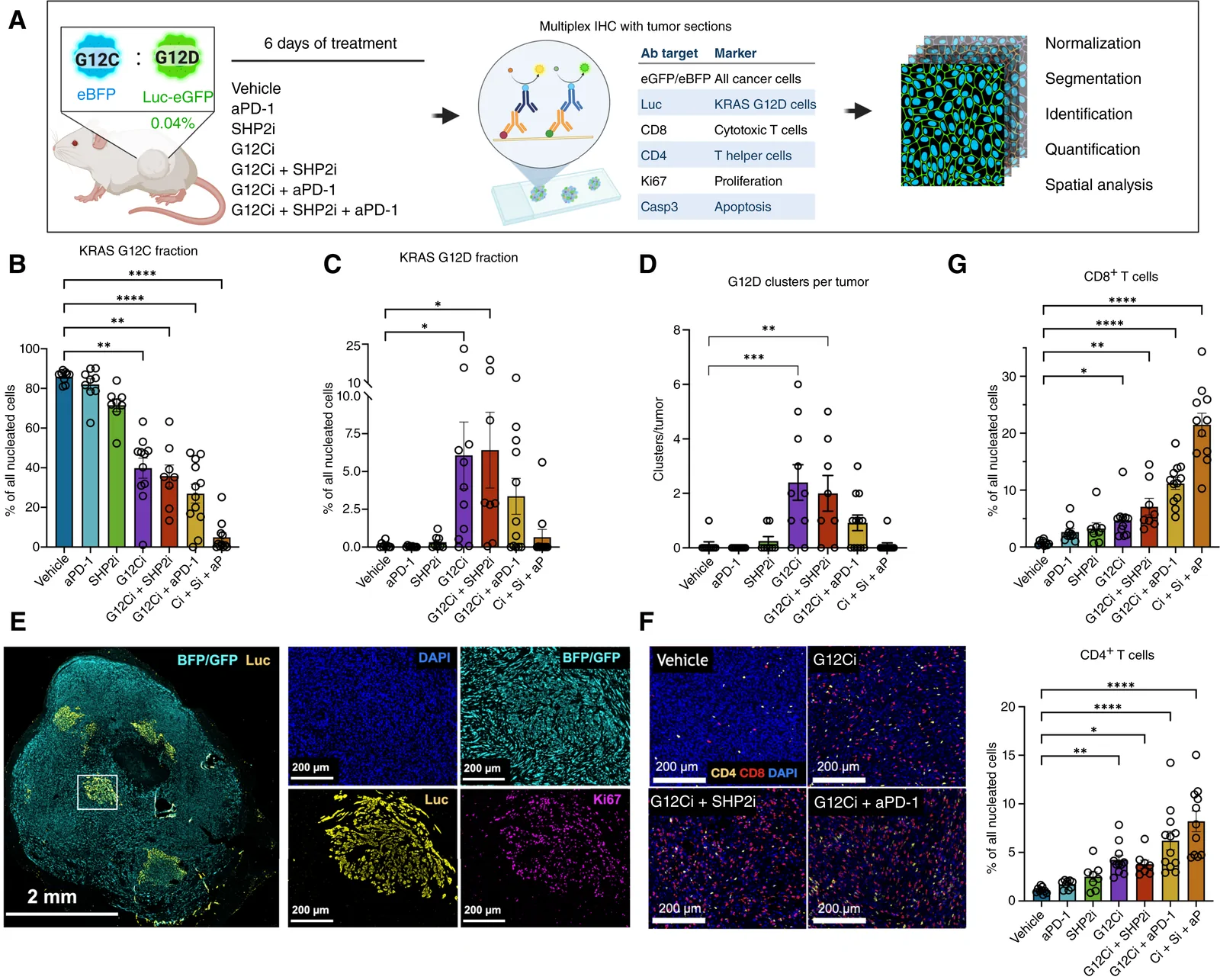
G12Ci treatment increases the proportion of KRAS G12D cells and T-cell infiltration. **A,** Schematic: Mixed subcutaneous tumors (BFP KRAS G12C cells plus 0.04% Luc-eGFP KRAS G12D cells) were treated for 6 days with RMC-4998 (G12Ci) ± RMC-4550 (SHP2i) and/or anti–PD-1 (aPD-1). After treatment, tumors were fixed, and multiplex immunofluorescence (IF) was performed. Each dot represents one tumor; mean ± SD. One-way ANOVA between vehicle and treatments was performed. **B,** Fraction of KRAS G12C cells of all nucleated cells. Ci + Si + aP, G12Ci + SHP2i+ anti–PD-1. **C,** Fraction of KRAS G12D cells of all nucleated cells. **D,** Number of KRAS G12D clusters per tumor section, defined as cell aggregates with more than 100 G12D cells. **E,** Representative tumor section of G12Ci-treated tumor (left) with a region of interest of a proliferating KPAR1.3 KRAS G12D cluster (right). Markers: BFP/GFP as a pan-cancer cell marker, luciferase (Luc) demarcating KRAS-G12D cells, and Ki67 indicating proliferation. **F,** Representative merged images from multiplex IF staining showing CD4^+^ T cells (yellow), CD8^+^ T cells (red), and DAPI; scale bar, 200 μm. **G,** Fractions of CD8^+^ (top) and CD4^+^ (bottom) T cells of all nucleated. *, *P* < 0.05; **, *P* < 0.01; ***, *P* < 0.001; ****, *P* < 0.0001.

To visualize the spatial distribution of the G12Ci-sensitive and -resistant cells as well as T-cell infiltration, we performed multiplexed immunofluorescence stainings, followed by segmentation and spatial analysis (Fig. 3A). Tumors with a minor resistant subpopulation were treated for 6 days, coinciding with the increase in bioluminescence (Fig. 2E). To avoid the region of interest sampling bias, we developed a novel, machine learning–based, high-throughput pipeline for cell segmentation and quantification (Supplementary Fig. S7).

As expected, cancer cell quantification revealed a reduction in KRAS G12C cells and increased the KRAS G12D fraction across treatments (Fig. 3B and C; Supplementary Fig. S8A). At this earlier timepoint, differences between G12Ci monotherapy and double combinations were limited. In contrast, the triple combination effectively eliminated both populations, consistent with the outcomes observed in the survival experiment. Spatial analysis revealed that G12Ci-treated tumors, either as monotherapy or in the double combinations, contained distinctive clusters of KRAS G12D cells, suggesting clonal expansion of the G12Ci-resistant subpopulation (Fig. 3D and E; Supplementary Fig. S8B). Additionally, the majority of KRAS G12D cells were Ki67 positive (∼60%), in contrast to KRAS G12C cells (∼20%), confirming an increased proliferative activity in the resistant subpopulation (Fig. 3E; Supplementary Fig. S8C). Interestingly, the proliferative and spatial patterns were similar between monotherapy and double combinations, suggesting that immune-mediated control may occur at later timepoints.

Spatial analysis also revealed increased infiltration of CD4^+^ and CD8^+^ T cells following G12Ci treatment, further enhanced by combination therapies, consistent with previous studies (Fig. 3F and G; refs. 24, 32). This was accompanied by an increase in Ki67-positive T cells (Supplementary Fig. S8D). Interestingly, combination treatments also increased cleaved Casp3–positive CD8^+^ T cells. Although these remain an extremely small fraction of the total, this might reflect activation-induced cell death (Supplementary Fig. S8E). Increased T-cell density correlated with depletion of G12Ci-sensitive cells, an effect most pronounced in triple combination–treated tumors, in which T cells exceeded cancer cells (Supplementary Fig. S8F).

Because G12Ci-resistant cell clusters are highly proliferative and unaffected by KRAS inhibition, we next investigated their impact on T-cell infiltration. Comparison of T-cell density inside versus outside resistant clusters showed similar CD8^+^ T-cell infiltration, indicating that treatments also result in a CD8^+^ T-cell enrichment inside the clusters compared with vehicle-treated tumors (Supplementary Fig. S8G). However, proliferative (Ki67-positive) CD8^+^ T cells were reduced within the clusters (Supplementary Fig. S8H). In contrast, CD4^+^ T cells did not show increased infiltration within clusters compared with vehicle-treated tumors, revealing a distinct spatial pattern (Supplementary Fig. S8I).

Overall, the spatial analysis indicates that the minor G12Ci-resistant subpopulation expands into highly proliferative clusters in response to G12Ci, whether given as monotherapy or in combination. Treatments also triggered a marked influx of T cells, which infiltrated both the G12Ci-sensitive tumor bulk and, to a lesser extent, the resistant clusters.

### Combination therapies increase inflammatory-associated signatures in G12Ci-resistant cells

**Figure 4.**
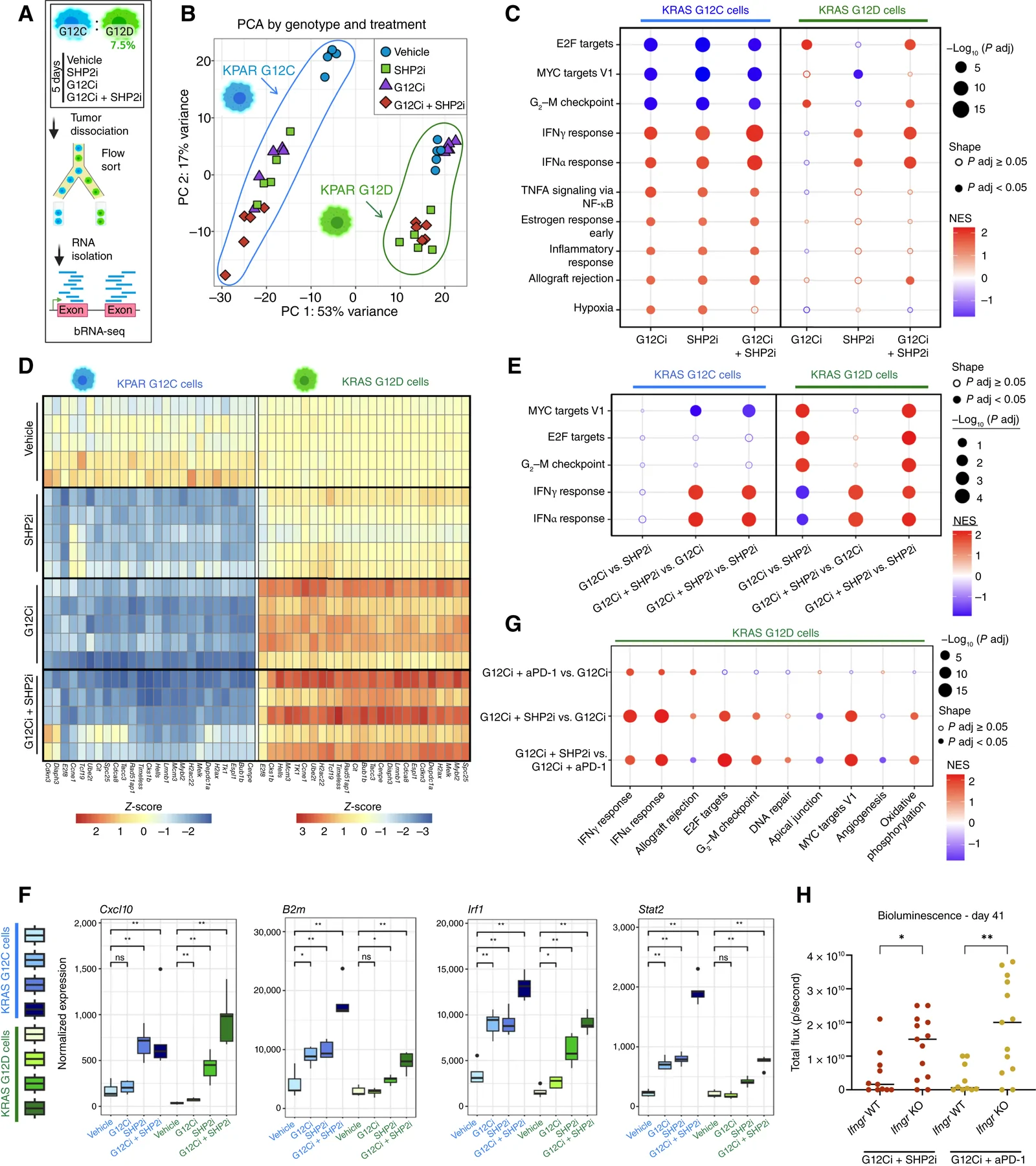
G12Ci-resistant subpopulation undergoes transcriptional changes in response to G12Ci and combinations. **A,** Schematic of the RNA-seq experiment. Mixed subcutaneous tumors (BFP KRAS G12C cells plus 7.5% Luc-eGFP KRAS G12D cells) were treated for 5 days with RMC-4998 (G12Ci) ± RMC-4550 (SHP2i). After treatment, cancer cells were sorted and bulk RNA-seq (bRNA-seq) was performed. **B,** Principal component analysis (PCA) across all samples and treatment groups; genotype of respective samples is indicated. PC, principal component. **C,** GSEA of top 10 differentially regulated Hallmark gene sets; vehicle set as baseline. NES, normalized enrichment score. **D,** Heatmaps for top 20 leading edge genes of the E2F target collection from the comparison between vehicle and G12Ci for KRAS G12C (left) and KRAS G12D cells (right). *Z*-scores normalized to the average of five vehicle samples (set to zero). **E,** GSEA of secondary treatment effects for KRAS G12C and KRAS G12D cancer cells. Second treatment on *x*-axis refers to baseline. Scoring as indicated in **C**. **F,** Expression of individual genes related to inflammatory response. Box plots indicate median and interquartile range. Statistical significance between vehicle- and inhibitor-treated samples was assessed using the pairwise Wilcoxon rank-sum tests (two-sided). **G,** Mixed subcutaneous tumors (BFP KRAS G12C cells plus 0.5% Luc-eGFP KRAS G12D cells) were treated for 6 days with RMC-4998 (G12Ci) ± RMC-4550 (SHP2i) or anti–PD-1 (aPD-1). KRAS G12D cells were sorted, and bulk RNA-seq was performed. GSEA of top 12 differentially regulated gene sets (MSigDB Hallmarks) for the comparison indicated in *x*-axis; the latter condition is used as the reference. Scoring as indicated in **C**. **H,** Mixed subcutaneous tumors with BFP KRAS G12C cells plus 0.04% of Luc-eGFP KRAS G12D cells expressing *Ifngr* wild-type (WT) or KO were treated for 2 weeks with RMC-4998 plus RMC-4550 (G12Ci + SHP2i) or anti–PD-1 (G12Ci + aPD-1). Bioluminescence imaging indicating relative abundance of KRAS G12D cells 18 days after treatment withdrawn (day 41 from treatment start). One-way ANOVA comparing WT vs. KO. *, *P* < 0.05; **, *P* < 0.01; ns, not significant.

To investigate how treatments affect G12Ci-sensitive and -resistant cells in mixed tumors, we performed transcriptomic analysis of the two populations after 5 days of treatment with vehicle, G12Ci, SHP2i, or the combination. eBFP-KRAS-G12C and Luc-eGFP-KRAS-G12D cells were sorted based on their fluorescent tags and subjected to bulk RNA sequencing (RNA-seq; Fig. 4A). Principal component analysis revealed treatment-driven clustering in the KRAS G12D samples, suggesting transcriptional changes in response to treatment, despite not being directly targeted by G12Ci (Fig. 4B; Supplementary Fig. S9A and S9B).

GSEA revealed that G12Ci suppressed proliferation-associated signatures E2F targets and G_2_–M checkpoint in KRAS G12C cells but upregulated them in KRAS G12D cells (Fig. 4C; Supplementary Table S6). This opposing transcriptional response was supported by leading-edge gene heatmaps (Fig. 4D) and selected proliferation-related genes (Supplementary Fig. S9C). In contrast, SHP2 inhibition downregulated Myc targets in both genotypes, likely reflecting tumor cell–intrinsic effects of SHP2 inhibition. In fact, although SHP2i alone did not inhibit KRAS G12D cell growth (Fig. 2A), it still suppressed the expression of RAS target genes both *in vitro* and *in vivo*; however, this effect was smaller in KRAS G12D cells (Supplementary Fig. S9D and S9E).

Consistent with previous reports (24), inflammatory response–associated signatures, including IFNγ and IFNα signaling pathways, were upregulated in KRAS G12C cells following treatments (Fig. 4C). Notably, these pathways were also induced in KRAS G12D cells by SHP2i or combination therapy. *In vitro* data indicated that SHP2i elicited a weaker IFN response in KRAS G12D cells (Supplementary Fig. S10A), consistent with their reduced sensitivity to SHP2 inhibition. This suggests that the observed IFN pathway activation in KRAS G12D cells is driven primarily by extrinsic signals from the TME. Moreover, the addition of G12Ci, despite not directly targeting KRAS G12D cells, further enhanced IFN pathway activation in these cells (Fig. 4E), supporting a role for non–cancer cell–intrinsic factors in shaping the transcriptional response of KRAS G12D cells. Importantly, this included upregulation of genes involved in antigen presentation and T-cell recruitment that could enhance immune recognition of the G12Ci-resistant cells (Fig. 4F; Supplementary Fig. S10B).

To further assess combination effects on KRAS G12D subpopulations, we performed a second RNA-seq experiment (Supplementary Fig. S10C). GSEA confirmed that adding SHP2i to G12Ci induced substantial transcriptional changes in KRAS G12D cells, including upregulation of IFN response signatures (Fig. 4G; Supplementary Fig. S10D and S10E; Supplementary Table S7). In contrast, the transcriptional profile of G12Ci plus anti–PD-1 clustered closer to G12Ci alone (Supplementary Fig. S10E). Nevertheless, this combination also enhanced IFN signaling in KRAS G12D cells, although to a lower extent than the combination with SHP2i (Fig. 4G).

Given that tumor cell–intrinsic IFNγ is required for antitumor immune responses (43), we generated *Ifngr* KO Luc-eGFP KRAS G12D cells to assess whether this pathway contributes to the clearance of G12Ci-resistant subpopulations. Bioluminescence scans revealed that the IFNγ-insensitive, G12Ci-resistant subpopulation expanded more rapidly (Supplementary Fig. S10F). This was validated at endpoint (Fig. 4H), indicating that IFN responses contribute to the immune-dependent control of the resistant subpopulation.

In summary, these results further corroborate an enhanced proliferative signature in the G12Ci-resistant subpopulation following G12Ci, alone or in combination. However, combination therapies also enhanced inflammatory responses, including IFN responses and antigen presentation, not only in G12Ci-sensitive cells but also in the resistant subpopulation. This immunomodulatory effect may be indicative of a pro-inflammatory TME induced by the combination therapies and may contribute to the immune-mediated elimination of the treatment-resistant cells.

### Immunophenotyping reveals treatment-driven shift toward a more inflamed TME

**Figure 5.**
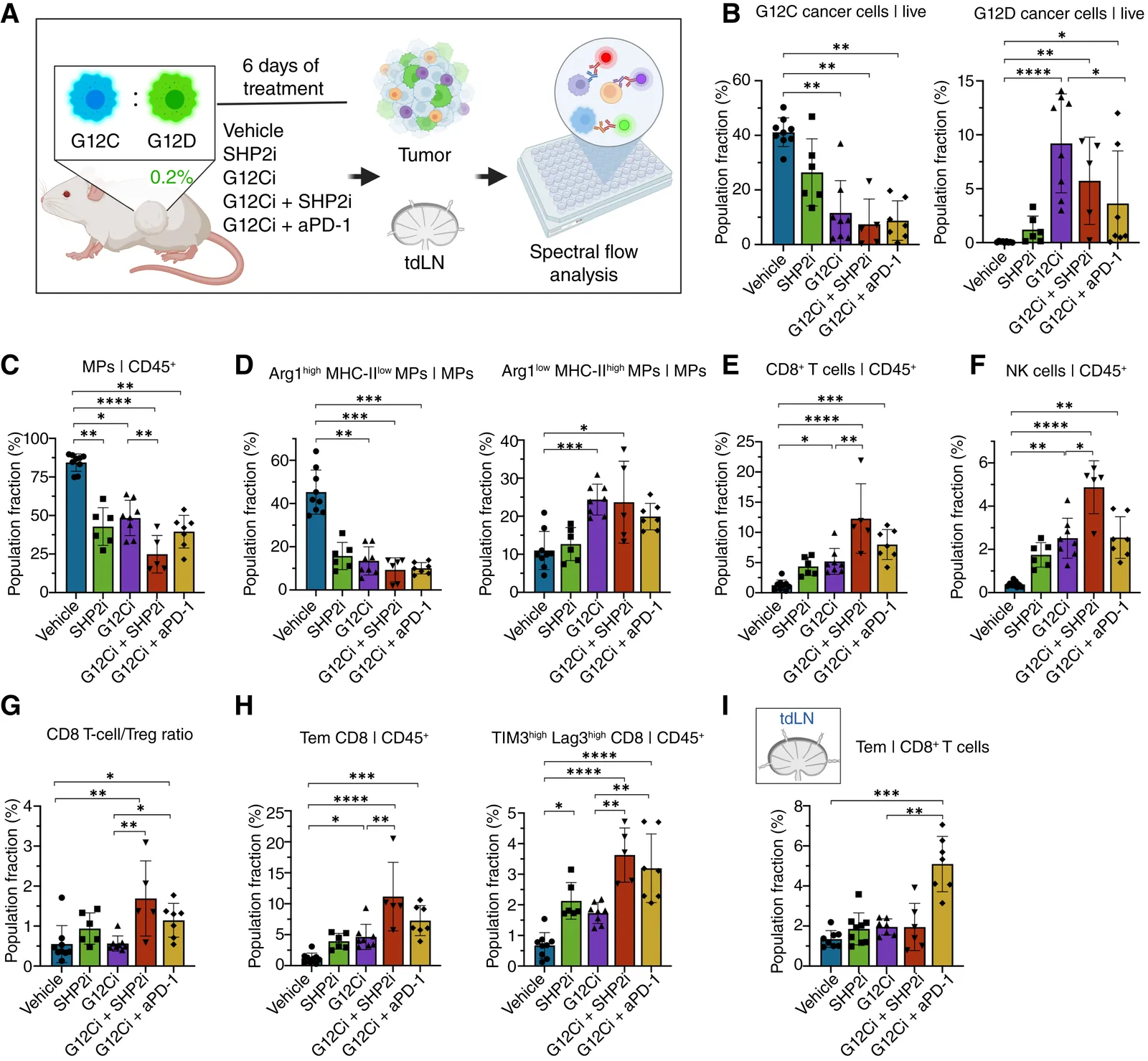
Treatments modulate the immune TME and increase inflammation. **A,** Schematic: Mixed subcutaneous tumors (BFP KRAS G12C cells plus 0.2% Luc-eGFP KRAS G12D cells) were treated for 6 days with RMC-4998 (G12Ci) ± RMC-4550 (SHP2i) or anti–PD-1 (aPD-1). Flow cytometry of tumors and tdLNs was performed. For all subsequent plots, each dot represents one individual tumor; mean ± SD; one-way ANOVA Kruskal–Wallis test comparing vehicle with each treatment or G12Ci with combination treatments (only significant comparisons). **B,** Fraction of KRAS G12C (left) and KRAS G12D (right) cells of all live cells. **C,** Fraction of macrophages (MP) of all immune cells (CD45^+^). **D,** Macrophage polarization: fraction of Arg1^high^ MHC-II^low^ (left) and Arg1^low^ MHC-II^high^ (right) MPs of all MPs. **E,** Fraction of CD8^+^ T cells of CD45^+^ cells. **F,** Fraction of NK cells of CD45^+^ cells. **G,** CD8^+^ T-cell/Treg (CD4^+^ Foxp3^+^ T cells) ratio. **H,** Fraction of Tem (CD44^+^/CD62L^−^; left) and TIM3^high^ Lag3^high^ (right) CD8^+^ T cells of CD45^+^ cells. **I,** In tdLN, fraction of Tem (CD44^+^/CD62L^−^) CD8^+^ T cells of all CD8^+^ T cells. *, *P* < 0.05, **, *P* < 0.01: ***, *P* < 0.001, ***, *P* < 0.0001.

Given the increased pro-inflammatory signatures in KRAS G12C and KRAS G12D cells (Fig. 4), along with the higher T-cell infiltration observed in the combination-treated tumors (Fig. 3), we next immunophenotyped the tumors to investigate how the different therapies remodel the immune TME (Fig. 5A). Consistent with our previous findings, 6-day treatment with G12Ci as monotherapy or in combination led to a reduction in the proportion of KRAS G12C cells, accompanied by an increase in KRAS G12D cells (Fig. 5B), whereas engraftment ratios were maintained in vehicle-treated mice (Supplementary Fig. S11A). Additionally, cancer cells upregulated MHC-I and PD-L1 expression following SHP2i or combination therapy, consistent with the enhanced inflammatory signatures observed under these conditions (Supplementary Fig. S11B).

Analysis of the immune cell compartment revealed that treatment resulted in a profound remodeling of the TME across multiple immune cell subpopulations (Supplementary Fig. S11C). Within the innate immune cell compartment, a significant reduction in macrophages was observed across all treatment groups (Fig. 5C). This was primarily driven by the decrease in Arg1^high^ MHC-II^low^ macrophages, which have been linked to more protumorigenic functions, whereas the percentage of more pro-inflammatory Arg1^low^ MHC-II^high^ macrophages increased (Fig. 5D). In addition, SHP2 inhibition also led to a significant increase in expression of the T-cell costimulatory transmembrane protein CD86 across all macrophages (Supplementary Fig. S11D), suggesting enhanced immune activation and highlighting additional effects of SHP2 in the myeloid compartment.

Analysis of the lymphoid compartment corroborated the increase in CD8^+^ and CD4^+^ T helper cell infiltration (Fig. 5E; Supplementary Fig. S11E). This was accompanied by an increase in NK and NKT cells, particularly pronounced after SHP2i combination (Fig. 5F; Supplementary Fig. S11F). In parallel, regulatory T cells (Treg) increased across treatment groups (Supplementary Fig. S11G), potentially dampening antitumor immune responses. However, the CD8^+^ T-cell/Treg ratio, generally considered a favorable prognostic indicator, was elevated in the combination therapies (Fig. 5G). Further characterization of CD8^+^ T cells revealed increased effector memory T-cell (Tem) and exhausted T-cell subsets (Fig. 5H). However, when expressed as percentages within the CD8^+^ compartment, these increases were not apparent (Supplementary Fig. S11H), suggesting that the expansion of activated and exhausted CD8^+^ T cells primarily reflects increased T-cell influx.

To assess whether treatment-induced effects extended beyond the tumor site, we also performed immunophenotyping of inguinal tdLNs adjacent to the subcutaneous tumors. This analysis revealed a significant increase in CD8^+^ Tem cells in the mice treated with G12Ci plus anti–PD-1, suggesting a potential systemic enhancement of T-cell priming (Fig. 5I). This effect was not observed in the G12Ci plus SHP2i combination, although we cannot exclude systemic immune modulation of this combination at later timepoints.

In summary, these results demonstrate that G12Ci, both as monotherapy and in combination, induces a profound remodeling of the immune TME, resulting in an enhancement of antitumor immunity that could facilitate the immune-mediated targeting not only of G12Ci-sensitive cells but also of resistant subpopulations.

### Adaptive immunity is required for durable responses and control of G12Ci-resistant subpopulations

**Figure 6.**
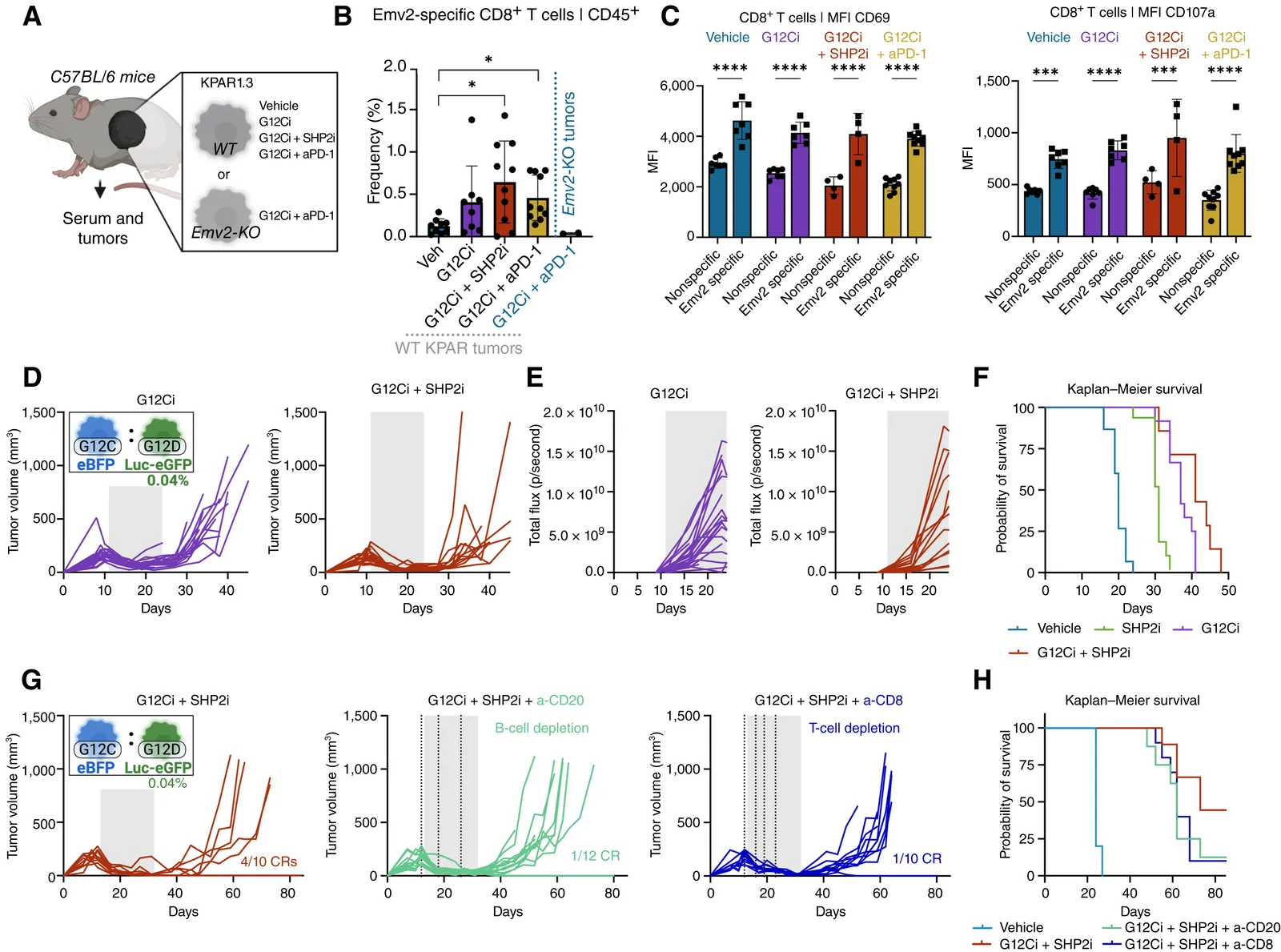
Adaptive immunity is required for durable responses. **A,** Experimental design for **B** and **C**. KRAS G12C KPAR1.3 wild-type (WT) or *Emv2*-KO tumors were treated for 6 days with RMC-4998 (100 mg/kg; G12Ci) ± RMC-4550 (SHP2i) or anti–PD-1 (aPD-1). **B,** Fraction of Emv2-specific CD8^+^ T cells of all immune cells (CD45^+^). Each dot represents one individual tumor; mean ± SD; one-way ANOVA Kruskal–Wallis test comparing vehicle with each treatment (only significant comparisons). **C,** Comparison of activation marker expression between Emv2-specific vs. non-specific CD8^+^ T cells for CD69 (left) and CD107a (right). **D–F,** Mixed subcutaneous tumors (BFP KRAS G12C cells plus 0.04% Luc-eGFP KRAS G12D cells) implanted in *Rag1*^−/−^ GH mice were treated for 2 weeks with RMC-4998 (G12Ci) and/or RMC-4550 (SHP2i). Graphs combine the replicate results of *n* = 2. Tumor volume over time of individual tumors (**D**). Bioluminescence scans indicating the relative abundance of KRAS G12D cells (**E**). Probability of survival, stratified by treatment group (**F**). **G** and **H,** Mixed subcutaneous tumors (BFP KRAS G12C cells plus 0.04% Luc-eGFP KRAS G12D cells) engrafted in immunocompetent GH mice were treated for 3 weeks with vehicle or RMC-4998 + RMC-4550 (G12Ci + SHP2i). Either B cells or CD8^+^ T cells were selectively depleted by administering anti-CD20 (a-CD20) or anti-CD8 (a-CD8) antibodies, respectively. Tumor growth over time for indicated treatment groups. Complete responders (CR) indicated (**G**). Probability of survival, stratified by treatment group (**H**). *, *P* < 0.05; ***, *P* < 0.001, ****, *P* < 0.0001.

To assess whether there is also an influx of tumor antigen–specific T cells, we used the known major tumor-associated antigen (TAA) in KPAR1.3 tumors, the Emv2 endogenous retroviral envelope protein (28). Using a synthetic tetramer containing the CD8-specific epitope from Emv2, treatments increased influx of Emv2-specific CD8^+^ T cells when expressed as a proportion of total immune cells but not as the percentage of CD8^+^ T cells (Fig. 6A and B; Supplementary Fig. S12A). This was accompanied by an additional influx of CD8^+^ T cells not specific for Emv2 (Supplementary Fig. S12B). Therefore, the abundance of Emv2-reactive T cells seems to track the overall increase in T-cell infiltration, suggesting that their influx into the tumors may occur through the same mechanisms, likely cytokine driven. Notably, compared with the bulk T-cell population, the Emv2-reactive T cells showed increased expression of the early activation marker CD69 and the cytotoxic degranulation marker CD107a, suggesting a more activated phenotype (Fig. 6C).

To determine whether a primed adaptive immune response is essential for eliminating G12Ci-resistant subpopulations and achieving durable tumor control, we generated albino *Rag1*^−/−^ GH mice, lacking functional T and B cells. Although initial tumor growth responses were comparable with immunocompetent mice (Fig. 2C and D), all tumors relapsed following treatment withdrawal and no complete responses occurred (Fig. 6D; Supplementary Fig. S12C). Moreover, bioluminescence imaging revealed that, unlike in immunocompetent mice (Fig. 2E), the combination treatment failed to control the KRAS G12D subpopulation, which proliferated similarly to tumors receiving G12Ci monotherapy (Fig. 6E). Interestingly, a higher proliferation rate compared with vehicle was also observed in mice treated with SHP2i alone (Supplementary Fig. S12D). Differences in long-term responses were also evident when visualizing survival probabilities (Figs. 6F vs. 2D). These findings indicate that adaptive immunity is required to control the growth of G12Ci-resistant subpopulations and achieve durable therapeutic responses.

To dissect the relative contributions of T and B cells in mediating this immune control, we performed depletion experiments in immunocompetent mice. Interestingly, depletion of either CD8^+^ T cells or B cells reduced, but did not eliminate, complete responses to KRAS G12C plus SHP2 inhibition (Fig. 6G and H), suggesting that both cell types contribute to the elimination of KRAS G12D cells and are required for the generation of a durable antitumor immunity. B-cell infiltration was minor in this model (Supplementary Fig. S11C), so the impact of targeting B cells may reflect roles in promotion of T-cell function, antigen presentation, or the production of tumor-binding antibodies. Indeed, serum from mice bearing parental KPAR1.3 G12C tumors contained tumor-reactive antibodies that bound both KPAR1.3 G12C and G12D cells lines *in vitro* (Supplementary Fig. S12E). *Emv2*-KO KPAR1.3 cells confirmed antibody specificity for Emv2.

These data indicate that elimination of drug-resistant cancer cells after G12Ci combination therapy is dependent on an adaptive immune response, likely involving both T- and B-cell functions.

### Immune-mediated bystander killing eliminates G12Ci-resistant subpopulations independent of the resistance mechanism

**Figure 7.**
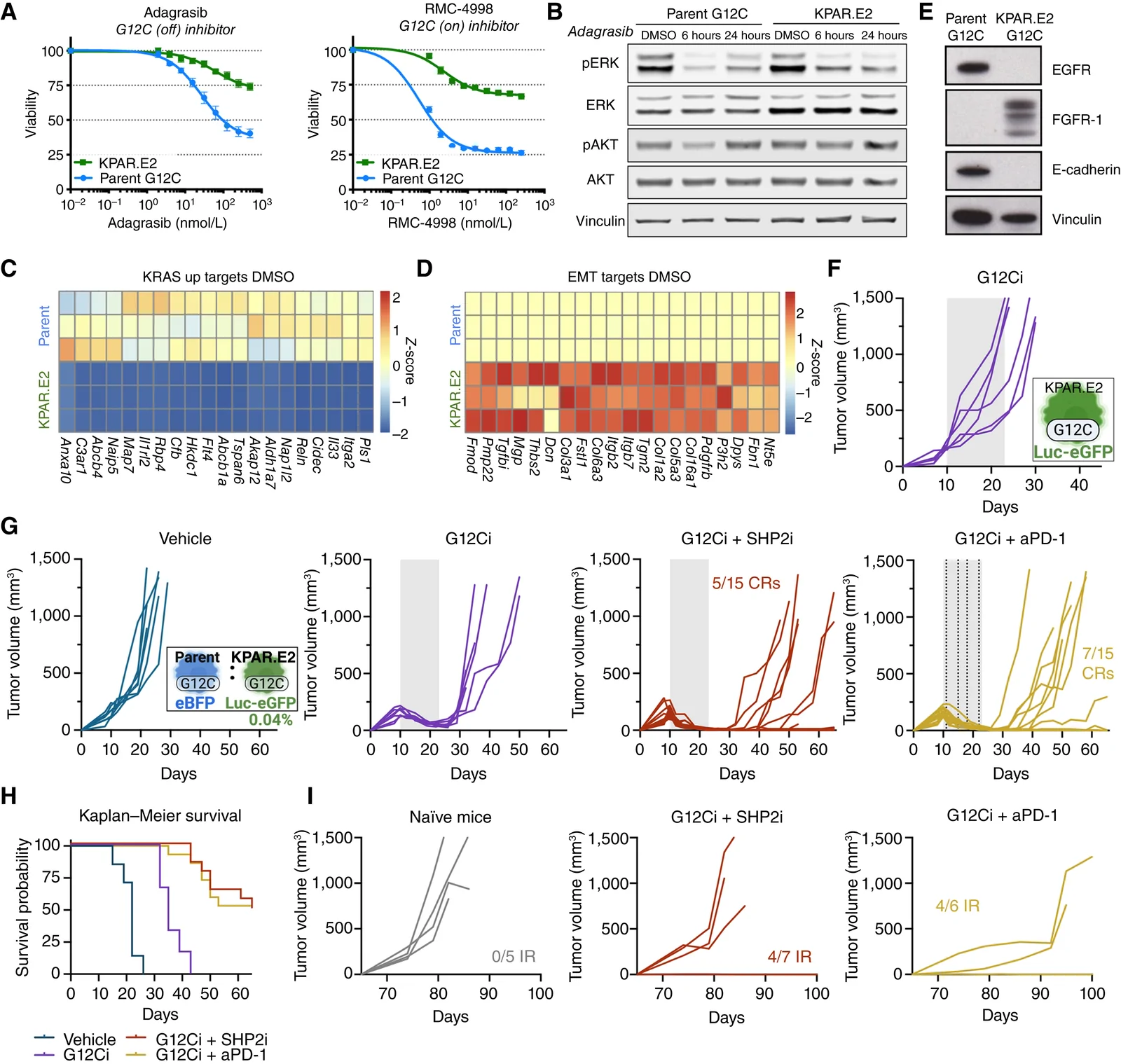
Immune rejection of mechanistically distinct G12Ci-resistant tumor subpopulations. **A,** Viability assays of KPAR.E2 cells and parental KPAR1.3 KRAS G12C cells (parent G12C). Cells were treated for 72 hours with the G12Cis adagrasib and RMC-4998. Mean ± SEM; *n* = 3. **B,** Western blots of cells treated at different timepoints with 100 nmol/L adagrasib. **C** and **D,** RNA-seq analysis comparing gene expression at baseline (DMSO-treated samples) between the KPAR1.3 KRAS G12C cell line (parent) and KPAR.E2 cells. Heatmaps for leading-edge genes from GSEA (MSigDB Hallmarks; KRAS up (**C**) and EMT signatures (**D**). *Z*-scores normalized to parent group mean. **E,** Western blots comparing basal protein expression between parental KPAR1.3 and KPAR.E2 cells. **F,** Tumor growth of Luc-eGFP KPAR.E2 subcutaneous tumors treated for 2 weeks with RMC-4998 (G12Ci). Growth of vehicle-treated mice is shown in Supplementary Fig. S15A. **G** and **H,** Mixed subcutaneous tumors (BFP KRAS G12C cells plus 0.04% Luc-eGFP-KPAR.E2 cells) were treated for 2 weeks with RMC-4998 (G12Ci) ± RMC-4550 (SHP2i) or anti–PD-1 (aPD-1). Growth of individual tumors. Complete responders (CR) indicated (**G**). **H,** Survival probability across treatment groups; cut-off value: 500 mm^3^(**H**). **I,** Rechallenge of complete responders (CRs) from **G** with untraced KPAR.E2 cells compared with naïve control mice. Immune-rejected (IR) tumors are indicated as the fraction of all CRs. Graph titles indicate the treatment that the primary tumor received.

The preclinical murine model system used so far does not reflect nongenetic or adaptive resistance mechanisms, as KRAS G12D cells have not undergone selective pressure from prior G12Ci exposure. To explore whether combination therapies could induce immune-mediated bystander elimination of minor resistant subpopulations arising through alternative mechanisms, we generated a G12Ci-resistant tumor by treating KPAR1.3 KRAS G12C orthotopic tumors *in vivo* with adagrasib until resistance emerged (Supplementary Fig. S13A). Clonal cell lines were derived from resistant tumors, and a representative subclone, KPAR.E2, was selected. Sanger sequencing of the *Kras* transcript confirmed that the *Kras* G12C mutation was retained, and no additional alterations were detected (Supplementary Fig. S13B). Viability assays confirmed that KPAR.E2 cells were resistant to both inactive- and active-state KRAS G12C inhibitors (Fig. 7A), as well as resistant to MEK inhibition, while remaining sensitive to PI3K inhibition (Supplementary Fig. S13C). *In vivo*, KPAR.E2-derived subcutaneous tumors progressed on adagrasib treatment, although with a modest reduction in the growth rate (Supplementary Fig. S13D and S13E).

ERK activity was suppressed in response to G12Ci treatment, and unlike the parental cell line KPAR.E2 cells did not exhibit a rebound in ERK phosphorylation at 24 hours, a well-described compensatory mechanism to KRAS inhibition (16), indicating that resistance in KPAR.E2 was not driven by RAS–MAPK reactivation (Fig. 7B; Supplementary Fig. S13F). To further characterize the mechanisms of resistance, we performed RNA-seq on KPAR.E2 and parental KPAR1.3 cells (Supplementary Fig. S14A). KRAS G12C inhibition suppressed RAS pathway transcriptional targets in both cell lines (Supplementary Fig. S14B). However, GSEA showed lower basal KRAS signaling in KPAR.E2 cells (Fig. 7C; Supplementary Fig. S14C). In addition, GSEA also revealed significantly enriched EMT-associated signatures in KPAR.E2 compared with parental cells (Fig. 7D; Supplementary Fig. S14D), further supported by the upregulation of canonical EMT markers (Supplementary Fig. S14E). EMT has previously been associated with reduced KRAS dependency (44) and resistance to KRAS and other MAPK pathway inhibitors (17, 45). Moreover, in line with previous findings, EMT was accompanied by rewiring of receptor tyrosine kinase expression; parental KPAR1.3 cells expressed EGFR, whereas resistant KPAR.E2 cells expressed FGFR-1 (Fig. 7E; refs. 46, 47). KPAR.E2 cells have thus undergone EMT and lost their dependency on RAS signaling, thereby conferring resistance to G12Ci.

Next, we transduced the KPAR.E2 cell line with the Luc-eGFP reporter to allow tracking when engrafted as a G12Ci-resistant subpopulation. Treatment of Luc-eGFP-KPAR.E2 tumors confirmed the resistance to RAS (on) G12C–selective inhibitor RMC-4998 (G12Ci; Fig. 7F) along with only modest response to anti–PD-1 and SHP2i treatments (Supplementary Fig. S15A and S15B). Next, eBFP KRAS G12C subcutaneous tumors containing a small fraction of Luc-eGFP-KPAR.E2 cells were engrafted. No CRs were observed with G12Ci as monotherapy, whereas both combination therapies led to several CRs despite the presence of the G12Ci-resistant subpopulation (Fig. 7G and H). Moreover, consistent with the previous model, bioluminescence imaging showed that combinations effectively controlled the outgrowth of the resistant cells (Supplementary Fig. S15C), and these therapies did not cause any weight loss (Supplementary Fig. S15D). Furthermore, CRs presented an enhanced immune memory compared with naïve mice after tumor rechallenge in the opposite flank with untraced KPAR.E2 cells (Fig. 7I), supporting the role of adaptive immunity in eliminating resistant cells and establishing long-term immune protection.

These data show that the immune-mediated bystander killing of drug-resistant cells is not limited to a single mechanism of resistance but can also be seen in a setting where tumor cells have become resistant to therapy *in vivo*, raising the prospect of more general applicability of this therapeutic approach to tackling drug resistance.

## Discussion

Although initial clinical responses to recently approved RAS inhibitors are encouraging, it has become clear that acquired drug resistance is a widespread concern that severely limits the clinical utility of these agents. Rapid evolution of drug resistance is a serious issue affecting many targeted oncology drugs; this urgently needs to be addressed if these agents are to achieve lasting tumor control. In the case of KRAS G12C inhibitors, a wide spectrum of resistance mechanisms have been identified, probably arising as subclonal events, making it hard to design combination therapies that effectively block the signaling pathways driving the evolution of drug resistance.

We hypothesized that a better approach to tackling the rapid development of resistance might be to exploit the fact that KRAS inhibitors at least partially reverse the KRAS-mediated immunosuppression in order to promote immune-mediated attack on drug-resistant cells. Indeed, KRAS inhibitors increase antitumor immunity (4), potentially targeting both the bulk drug-sensitive tumor and also minor drug-resistant subclones sharing tumor antigens. In addition, the killing of large numbers of cancer cells by KRAS inhibitors likely increases the uptake of tumor antigens by antigen-presenting cells, providing an “*in situ*” vaccination effect to boost antitumor immunity.

To explore this approach to tackling the emergence of drug resistance, we developed a mouse cancer model that allows the study of the behavior of minor drug-resistant clones within a predominantly drug-sensitive tumor. Based on the KPAR1.3 lung cancer cell line (28), which forms tumors with a relatively inflamed TME, KRAS was engineered to either be sensitive (G12C) or insensitive (G12D) to G12Ci. Both populations can be distinguished by fluorescent markers and drug-resistant cells tracked *in vivo* by luciferase. Alternatively, we used G12Ci-resistant KRAS G12C cells that acquired resistance via EMT. Importantly, although these resistant cells are more divergent from the parental population, they still share the same TAA.

Using this system, G12Ci monotherapy led to rapid outgrowth of preexisting resistant populations, comparable with clinical responses. By 6 days of treatment, clusters of rapidly proliferating drug-resistant tumor cells emerged, coinciding with the bulk drug-sensitive tumor having lost proliferative capacity. The drug-resistant cells grow more rapidly when the tumor is under selective pressure from G12Ci, likely due to removal of competition from the bulk drug-sensitive population or the release of trophic factors from dying drug-sensitive cells that may promote the growth of drug-resistant cells (48).

The use of G12Ci monotherapy leads to profound remodeling of the TME, including influx of CD8^+^ T cells and NK cells and changes in the phenotypic state of macrophages. Despite this, G12Ci monotherapy is not sufficient to allow effective immune control of tumors with minor populations of drug-resistant cells. However, when an immune-targeted agent, such as anti–PD-1 or an SHP2i, is added to the G12Ci, complete tumor clearance is achieved in a high proportion of animals, although at a lower frequency than in G12C-only tumors (32, 49). This effect requires the presence of an adaptive immune response, involving both T and B cells. Although we cannot exclude a partial direct effect of SHP2 inhibition or anti–PD-1 therapy on KRAS G12D cells, we believe this primarily reflects immune-mediated bystander killing of the drug-resistant cells triggered by the elimination of drug-sensitive cells.

Mechanistically, it is likely that this immune-mediated killing of the drug-resistant cells involves targeting shared TAAs. In KPAR1.3 cells, we believe that the major TAA is the Emv2 envelope protein of the murine leukemia virus endogenous retrovirus (28), which can be recognized both by CD8^+^ and CD4^+^ T cells and also by antibodies. Emv2-specific CD8^+^ T cells increase with G12Ci and combination treatments, providing a possible mechanism for elimination of drug-resistant cells. Moreover, combination therapies increase the expression of MHC-I on the cancer cells, which would be expected to enhance antigen presentation and immune recognition.

Combination therapies also exhibit distinct immune-modulatory features. Immune profiling revealed greater T- and NK-cell infiltration with SHP2i, whereas the anti–PD-1 combination increased the proportion of Tem cells in the tdLNs, consistent with distinct immune dynamics. Further research will be needed to understand the full molecular details for the enhanced immune-mediated bystander killing of drug-resistant cells achieved by the combinations compared with G12Ci alone. However, the involvement of tumor antigen–specific T cells and IFN signaling is clear.

In terms of more immediate clinical translation, although G12Ci plus SHP2i potently promotes elimination of drug-resistant cells in this model, SHP2i is unlikely to enter routine clinical use because of significant toxicity concerns. On the other hand, anti–PD-1 is used widely, although early trials of combination therapies with first-generation G12Ci have been limited by significant toxicity (50). However, these toxicities are likely off-target effects, providing reason to be hopeful that combinations of anti–PD-1 with more specific, second-generation G12Ci, such as divarasib, olomorasib, and elironrasib, may allow the clinical exploration of this combination to control the development of drug-resistant disease in the relatively near future. The potential for this approach to have an impact on drug-resistant disease is likely to be best in the setting of tumors with an inflamed TME, comparable with the model used here. These findings also suggest that immune-enhancing therapies should be introduced up front, as an expansion of the resistant cells can reestablish KRAS-driven immunosuppressive mechanisms and dampen the immune response.

We believe that the model system described here firmly establishes the phenomenon of immune-mediated bystander killing of drug-resistant cells. It also provides a platform for determining optimal approaches to enhancing the strength and impact of this effect, which will be particularly important for more immune-evasive tumors that will not be so responsive to the combination of G12Ci with anti–PD-1. It is possible that additional immune-targeted agents may significantly potentiate this effect, particularly those that have previously induced durable responses when combined with KRAS inhibitors in immune-cold models. These include approaches aiming at depleting intratumoral Tregs, such as using CTLA-4– or CCR-8–depleting antibodies (51). Strategies targeting other aspects of the cancer immunity cycle could also be explored in this system, such as a CXCR1/2 inhibitor to limit immunosuppressive myeloid cells and 4-1BB agonistic antibody to enhance dendritic cells (52). In addition, incorporating pan-RAS inhibitors into the combination with G12Ci and anti–PD-1 may also provide additional therapeutic benefit (49). Alternatively, and more speculatively, the process of presentation of TAAs from dying cancer cells to immune effector cells may be another area that could be optimized, for example, by intervening in phagocytosis or making the mechanism of cell death more immunogenic. This may represent a paradigm for eliminating the development of disease that is resistant to oncogene-targeted agents through engagement of the immune system.

## Supplementary Material

Figure S1 Spectral flow cytometry gating strategy

Table S1 Western blot antibodies

Figure S2 Spectral flow cytometry gating strategy for Emv2 tetramer

Table S2 Real-time PCR primers

Figure S3 Generation of reporter-transduced KPAR1.3 cell lines

Table S3 Spectral flow cytometry staining panel

Figure S4 Characterisation of mixed KPAR 1.3 tumors

Table S4 Spectral flow cytometry staining panel for analysis of Emv2-specific T cells

Figure S5 Combination strategies to enhance treatment responses

Table S5 Predicted MHC-I binding affinities for eBFP peptides

Figure S6 Triple combination increases the generation of complete responders

Table S6 GSEA significant targets across all treatment groups

Figure S7 Outline of image analysis pipeline

Table S7 GSEA significant targets across all treatment groups

Figure S8 Spatial analysis of mixed KPAR1.3 subcutaneous tumors

Figure S9 RNAseq analysis of proliferation-associated tumor cell signatures

Figure S10 RNA-seq analysis of inflammation-associated tumor cell states

Figure S11 TME phenotyping via spectral flow cytometry

Figure S12 Adaptive immunity is required for durable responses

Figure S13 Establishment of G12Ci resistant KPAR.E2 cell line

Figure S14 RNA-seq analysis of KPAR.E2 cell line

Figure S15 Treatment of pure and mixed KPAR.E2 tumors

## Data Availability

The RNA-seq data for this study have been deposited in the European Nucleotide Archive at European Molecular Biology Laboratory's European Bioinformatics Institute under the accession numbers PRJEB101476, PRJEB101500, and PRJEB101588. Multiplex images will be available upon request. The remaining data are available within the article, Supplementary Information, or Source Data provided with this article.
