# Supplementary material for: KRAS Inhibitor–Induced Cancer Cell Death Enhances Sensitivity to Immune-Mediated Bystander Killing of Drug-Resistant Subclones
Source: Cancer Res. 2026 Jun 8;86(17):4334–51. doi: 10.1158/0008-5472.CAN-25-5693 (PMC13535323; doi:10.1158/0008-5472.CAN-25-5693)
Supplement: Supplementary file 1 [file can-25-5693_figure_s10_suppsf10.pdf]

Supplementary Figure S10

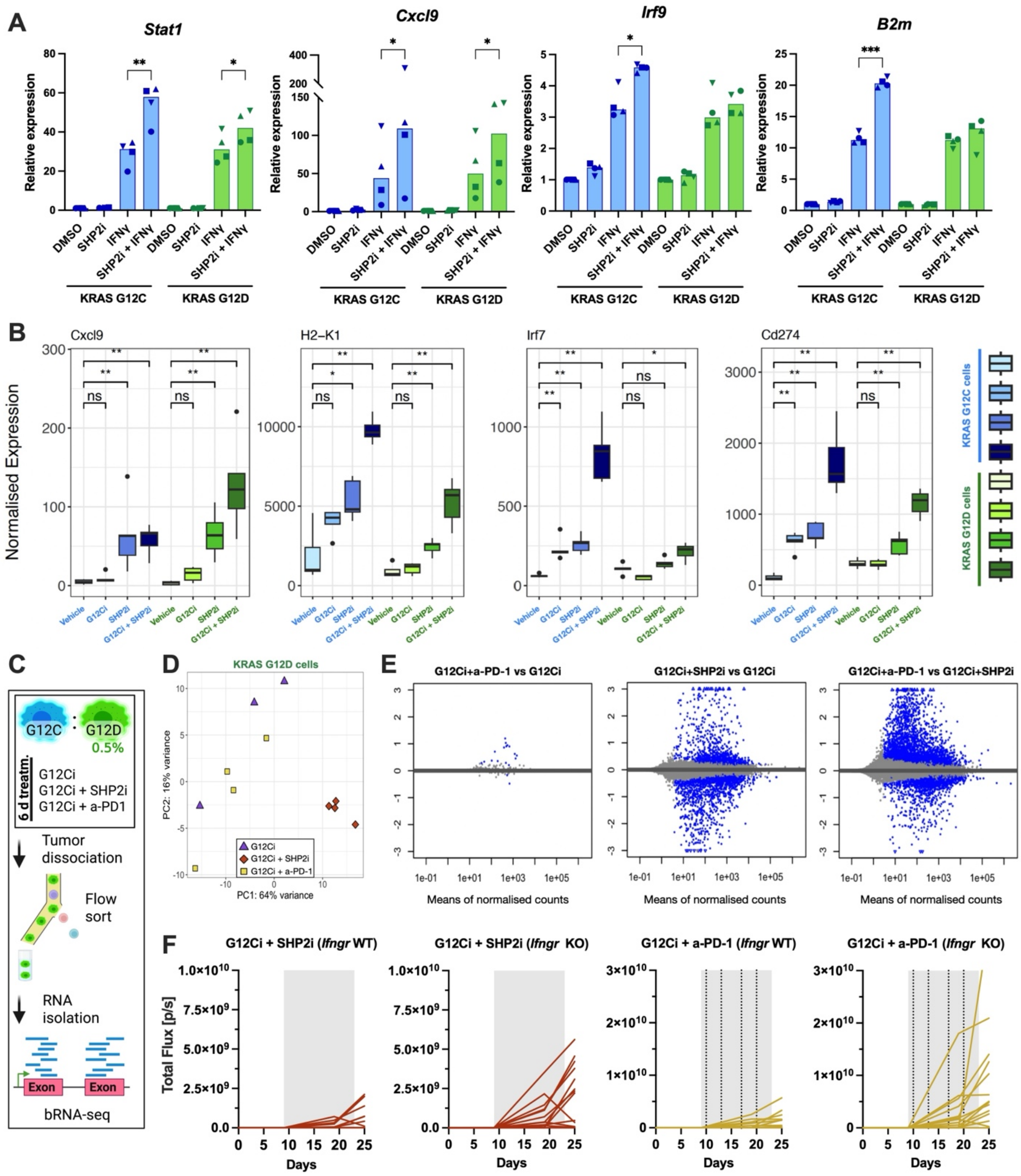

Supplementary Figure S10. RNA-seq analysis of inflammation-associated tumor cell states.

A) KRAS-G12C or G12D KPAR1.3 cells were treated in vitro for 24h with DMSO, 1µM RMC-4550 (SHP2i), IFNγ (10 ng/ml) or the combination of both and qPCR analysis of the indicated genes was performed. Mean ± SD, n=4 (indicated with different symbols), tr(n)=2. One-way ANOVA test was performed to compare IFN vs SHP2i + IFN conditions.

(B) Expression of individual genes related to interferon responses visualised from RNAseq as described in Figure 4A. Box plots indicate median, interquartile range. Whiskers represent the largest and smallest quartile and outliers are displayed as individual dots. Statistical significance between vehicle-treated and inhibitor-treated samples was assessed using pairwise Wilcoxon rank-sum tests (two-sideD).

C) Schematic of experimental workflow for panels (D-E). Mixed subcutaneous tumors were engrafted with BFP-KRAS-G12C cells plus 0.5% Luc-eGFP-KRAS-G12D cells. Mice were treated for five days with the RAS-G12C(ON) inhibitor RMC-4998 (100 mg/kg; C12Ci) with or without the SHP2 inhibitor RMC-4550 (30 mg/kg; SPH2i) or a-PD-1 (10 mg/kg). After treatment KRAS-G12D cells were sorted and bulk RNAseq was performed.

D) PCA analysis of KRAS-G12D mutant tumor cells across treatment groups.

E) MA-plots of secondary treatment effects, visualising the log2 fold changes over the mean expression of normalised gene counts. The later term in the respective plot titles is considered as “baseline” (set as 0 on x-axis). Data points on plot are coloured blue if padj ≤0.1. If data points fall out of the range of the defined y-axis they are plotted as triangles.

F) Bioluminescence scan data over time of mixed subcutaneous tumors engrafted with BFP-KRAS-G12C cells plus 0.04% of Luc-eGFP-KRAS-G12D cells expressing wild-type (WT) or knock-out (KO) *Ifngr* and treated for two weeks with 100 mg/kg RMC-4998 in combination with 30 mg/kg RMC-4550 (G12Ci + SHP2i) or 10 mg/kg anti-PD-1 (G12Ci + a-PD-1). Grey area indicates treatment period.
