# Supplementary material for: KRAS Inhibitor–Induced Cancer Cell Death Enhances Sensitivity to Immune-Mediated Bystander Killing of Drug-Resistant Subclones
Source: Cancer Res. 2026 Jun 8;86(17):4334–51. doi: 10.1158/0008-5472.CAN-25-5693 (PMC13535323; doi:10.1158/0008-5472.CAN-25-5693)
Supplement: Supplementary file 2 [file can-25-5693_figure_s11_suppsf11.pdf]

Supplementary Figure S11

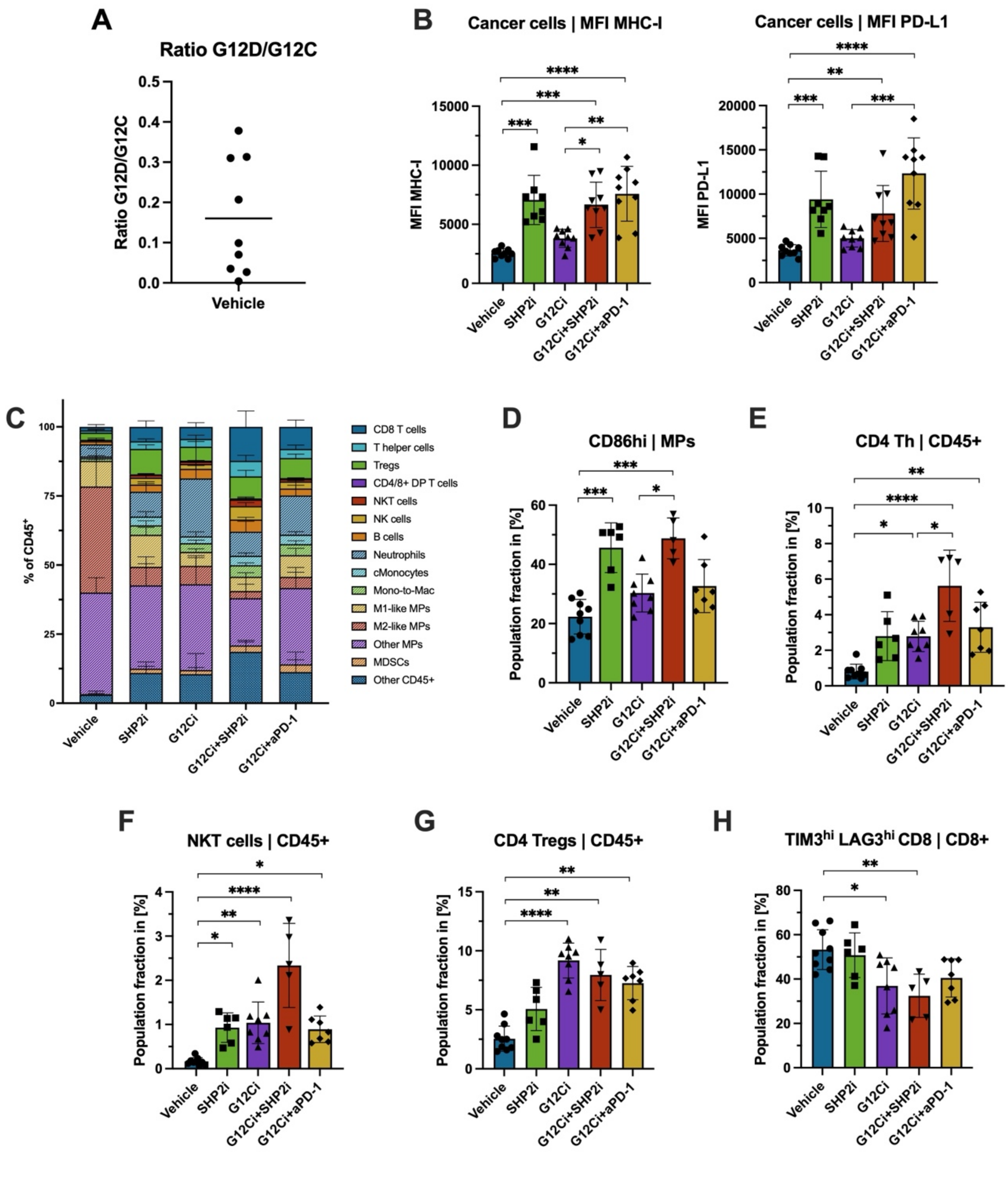

Supplementary Figure S11. TME phenotyping via spectral flow cytometry.
Mixed subcutaneous tumors were engrafted with BFP-KRAS-G12C cells plus 0.2% Luc-eGFP-KRAS-G12D cells. Mice were treated for six days with the RAS-G12C(ON) inhibitor RMC-4998 (100 mg/kg; G12Ci / Ci) with or without the SHP2 inhibitor RMC-4550 (30 mg/kg; SHP2i / Si) or a-PD-1 (10 mg/kg; P). After treatment flow cytometry of tumors was performed.
A) Ratio of KRAS-G12D / KRAS-G12D tumor cells out of all live tumor cells in vehicle-treated mice.

B) Surface expression (mean fluorescence intensity, MFI) of MHC-I (left) and PD-L1 (right) in cancer cells.
C) Mean percentages of $CD45^+$ immune cell sub-types by treatment groups, whiskers indicate SD within each population across individual samples. Abbreviations from top to bottom: 'Tregs': T regulatory cells'; '$CD8^+$ $CD4^+$ double-positive (DP) T cells', 'NKT': natural killer T cells; 'NK': natural killer cells; 'cMonocytes': classical ($CD64^+$) Monocytes; 'Mono-to-Mac': a population of monocytes in transition-phase to differentiating macrophages; 'M1-like MPs': $Arg1^{lo}$ $MHC\text{-}II^{hi}$ Macrophages; 'M2-like MPs': $Arg1^{hi}$ $MHC\text{-}II^{lo}$ Macrophages; 'Other MPs': Other Macrophages; 'MDSCs': myeloid derived suppressor cells.
(D-H) For all graphs, dots represent individual tumors; bars show mean + SD; one-way ANOVA Kruskal-Wallis test was performed, calculating pairwise comparison between vehicle and individual treatment groups or G12Ci to combination treatments; only significant comparisons are indicated.
D) Fraction of CD86 high cells as percentage of all macrophages (MPs).
E) Fraction of $CD4^+$ T helper cells out of all immune cells ($CD45^+$).
F) Fraction of NKT cells out of all $CD45^+$ cells.
G) Fraction of regulatory T cells (Tregs) out of all $CD45^+$ cells.
H) Fraction of terminally differentiated/exhausted ($TIM3^{hi}$/$LAG3^{hi}$) $CD8^+$ T cells out of all $CD8^+$ T cells.
