# Supplementary material for: KRAS Inhibitor–Induced Cancer Cell Death Enhances Sensitivity to Immune-Mediated Bystander Killing of Drug-Resistant Subclones
Source: Cancer Res. 2026 Jun 8;86(17):4334–51. doi: 10.1158/0008-5472.CAN-25-5693 (PMC13535323; doi:10.1158/0008-5472.CAN-25-5693)
Supplement: Supplementary file 3 [file can-25-5693_figure_s12_suppsf12.pdf]

Supplementary Figure S12

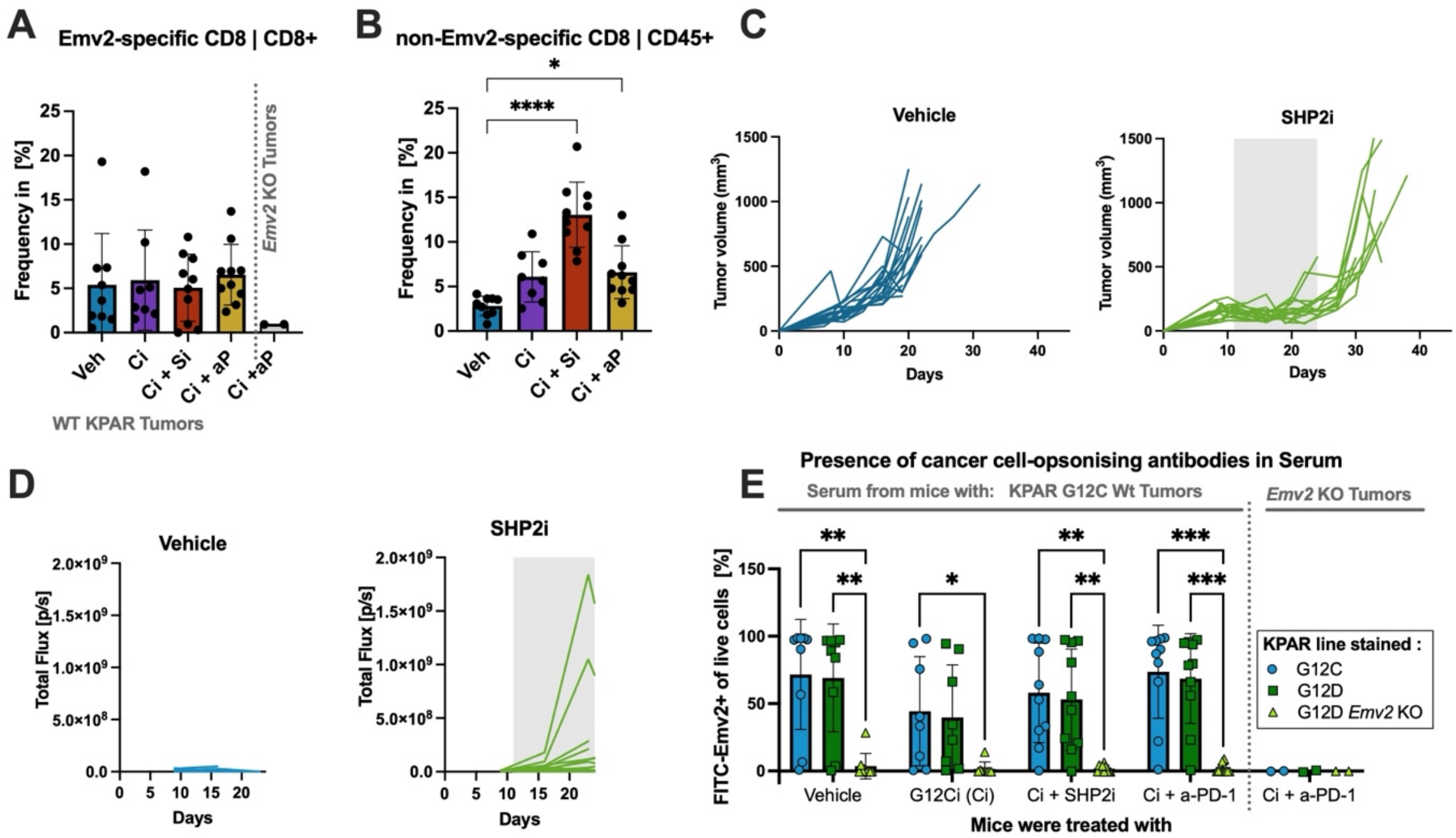

Supplementary Figure S12. Adaptive immunity is required for durable responses.
A-B) Mice with KRAS-G12C KPAR1.3 wild-type or *Emv2* knockout tumors were treated for six days with the RAS-G12C(ON) inhibitor RMC-4998 (100 mg/kg; G12Ci / Ci) with or without the SHP2 inhibitor RMC-4550 (30 mg/kg; SHP2i) or a-PD-1 (10 mg/kg; aP). A) Frequency of Emv2-specific $CD8^+$ T cells of all $CD8^+$ T cells. Additional control group with KPAR1.3 KRAS-G12D *Emv2*-KO tumors is shown. B) Frequency of non-Emv2-specific $CD8^+$ T cells of all immune cells ($CD45^+$). Each dot represents one individual tumor; bar graphs indicate mean ± SD; One-way ANOVA Kruskal-Wallis test comparing vehicle to each of the treated conditions; only significant comparisons are shown.
(C-D) Mixed subcutaneous tumors were engrafted with BFP-KRAS-G12C cells plus a fraction of 0.04% Luc-eGFP-KRAS-G12D cells in $Rag1^{-/-}$ GH-mice. Mice were treated for two weeks with vehicle or the SHP2 inhibitor RMC-4550 (30 mg/kg; SHP2i). C) Tumor growth of individual tumors across indicated treatment groups. Treatment interval is highlighted in grey. Additional treatment groups are shown in Figure 6D. D) Bioluminescence scans, indicating the relative abundance of Luc-eGFP-KRAS-G12D cells over time, measured in total flux, photons per second [p/s]. Additional treatment groups are shown in Figure 6E, with different axis scaling.
E) Median fluorescent intensity (MFI) of serum-stained un-transduced KPAR1.3 KRAS-G12C, G12D, or G12D *Emv2* KO tumors cancer cells, assessed via flow cytometric analysis. Serum was collected from mice treated as indicated in (A). All samples were normalised to the MFI of matching serum from obtained from one naïve mouse. Results are coloured by genotype and sorted by treatment group. Serum from a control group of mice harboring *Emv2* KO tumors was also included as additional negative control for epitope specificity. Each dot indicates serum from one mouse, mean + SEM. 2way ANOVA test was performed, between opsonised cell lines within each treatment condition, comparing serum samples

from KPAR1.3 G12D *Emv2* KO cells to the KPAR1.3 wt groups; only significant comparisons are indicated.
