# Supplementary material for: KRAS Inhibitor–Induced Cancer Cell Death Enhances Sensitivity to Immune-Mediated Bystander Killing of Drug-Resistant Subclones
Source: Cancer Res. 2026 Jun 8;86(17):4334–51. doi: 10.1158/0008-5472.CAN-25-5693 (PMC13535323; doi:10.1158/0008-5472.CAN-25-5693)
Supplement: Supplementary file 4 [file can-25-5693_figure_s13_suppsf13.pdf]

# Supplementary Figure S13

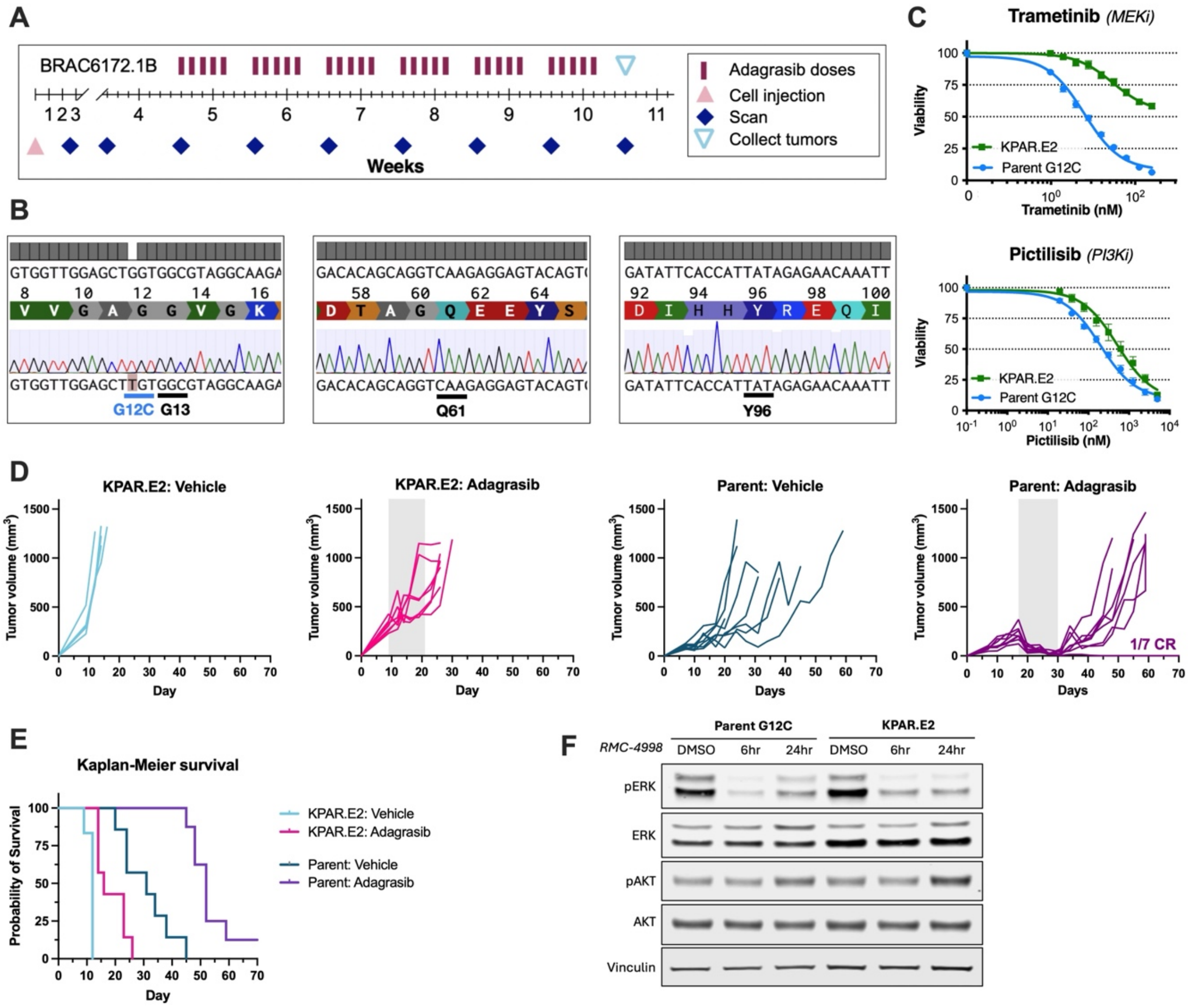


**Supplementary Figure S13. Establishment of G12Ci resistant KPAR.E2 cell line.**
A) Treatment scheme to generate adagrasib-resistant KPAR1.3 KRAS-G12C-derived lung tumors in immune-competent mice.
B) Sanger sequencing for KRAS transcript in KPAR.E2 cells (bottom) compared with reference sequence (top), highlighting the retained KRAS-G12C mutation as well as unaltered aminoacid sequence at other hotspot sites.
C) 72-hour viability assays for targeted inhibitors trametinib (MEKi) and pictilisib (PI3Ki) at indicated drug concentrations in parental KPAR1.3 KRAS-G12C cell line (Parent G12C) and G12Ci-resistant KPAR.E2 cells. Error bars indicate SEM with n=3.
D) Tumor growth of KPAR.E2 or KPAR1.3 KRAS-G12C (parent) subcutaneous tumors treated for 2 weeks (grey area) with either vehicle or the KRAS-G12Ci adagrasib (50 mg/kg). Complete responders (CR) indicated on respective plot.
E) Probability of survival for mice across all treatment groups from (D).
F) Western blot of cells treated at different time points with 100 nM RMC-4998.
