# Supplementary material for: KRAS Inhibitor–Induced Cancer Cell Death Enhances Sensitivity to Immune-Mediated Bystander Killing of Drug-Resistant Subclones
Source: Cancer Res. 2026 Jun 8;86(17):4334–51. doi: 10.1158/0008-5472.CAN-25-5693 (PMC13535323; doi:10.1158/0008-5472.CAN-25-5693)
Supplement: Supplementary file 5 [file can-25-5693_figure_s14_suppsf14.pdf]

Supplementary Figure S14

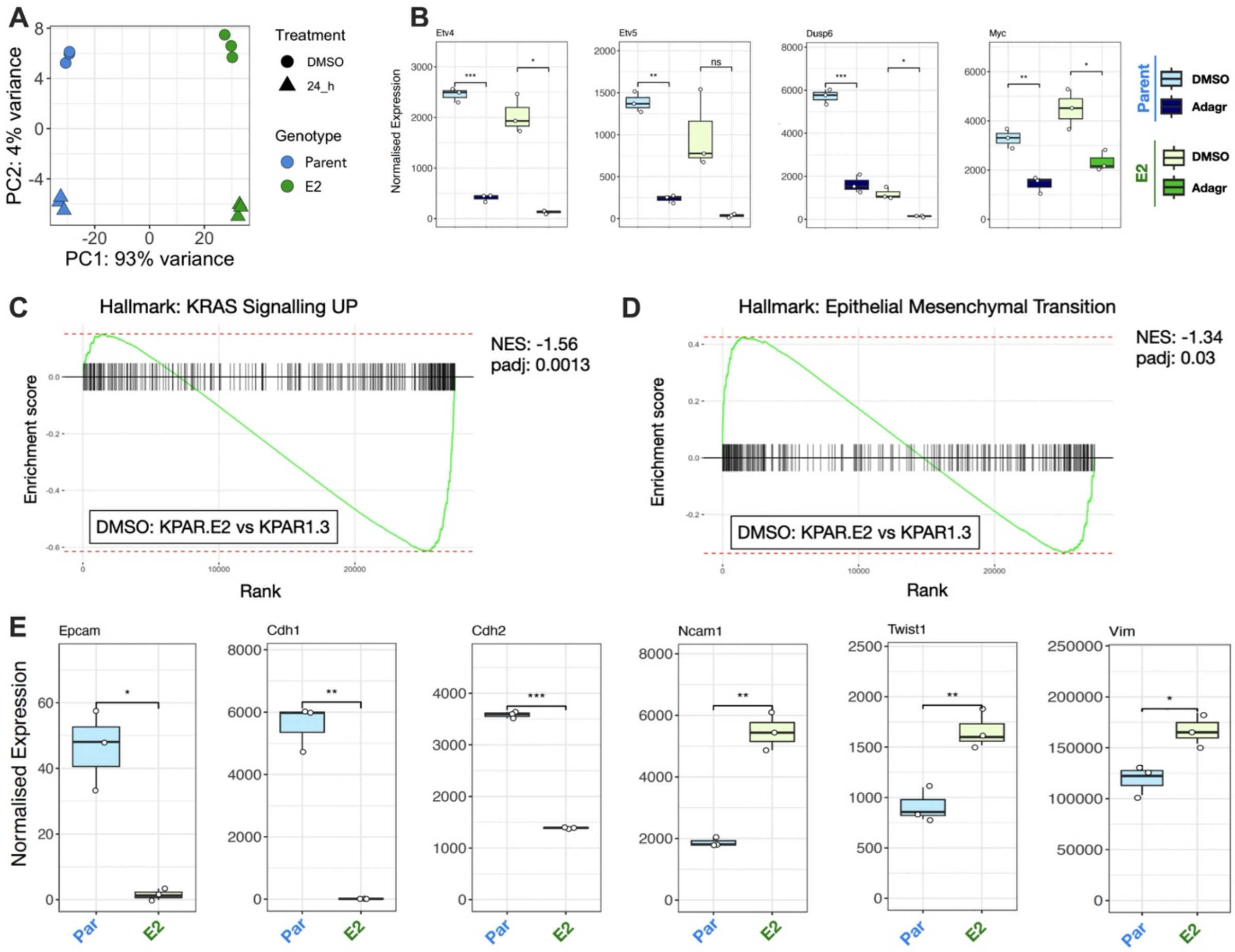

Supplementary Figure S14. RNA-seq analysis of KPAR.E2 cell line.
A) Principal component analysis (PCA) of RNA-sequencing samples of parental KPAR1.3 KRAS-G12C and KPAR.E2 cell lines treated either with DMSO or 100 nM adagrasib for 24h.
B) Expression of individual genes related to downstream transcriptional targets of KRAS signaling. Visualised as box plots, indicating median, interquartile range, whiskers represent largest and smallest quartile and outliers are displayed as individual dots.
C-D) Gene set enrichment analysis plots for indicated gene sets of the msigdb hallmarks collection, (C) KRAS Signaling UP and (D) Epithelial to mesenchymal transition.
E) Expression of individual genes related to EMT-associated genes. Visualised as box plots, indicating median, interquartile range, whiskers represent largest and smallest quartile and outliers are displayed as individual dots.
