# Supplementary material for: KRAS Inhibitor–Induced Cancer Cell Death Enhances Sensitivity to Immune-Mediated Bystander Killing of Drug-Resistant Subclones
Source: Cancer Res. 2026 Jun 8;86(17):4334–51. doi: 10.1158/0008-5472.CAN-25-5693 (PMC13535323; doi:10.1158/0008-5472.CAN-25-5693)
Supplement: Supplementary file 6 [file can-25-5693_figure_s15_suppsf15.pdf]

Supplementary Figure S15

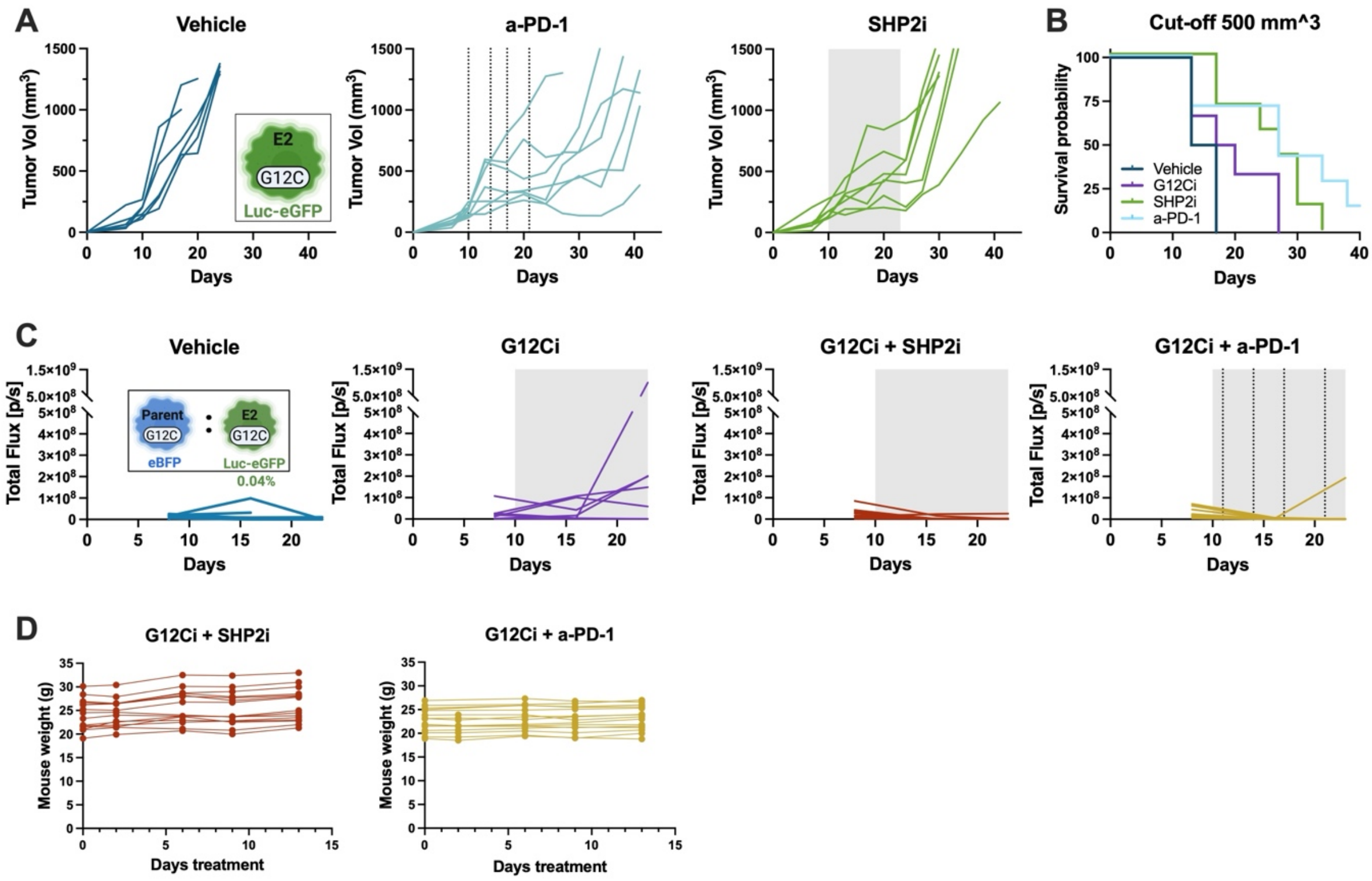

Supplementary Figure S15. Treatment of pure and mixed KPAR.E2 tumors.
A) Tumor growth over time of individual KPAR.E2-derived subcutaneous tumors for indicated treatment groups: vehicle, a-PD-1 (10 mg/kg) and SHP2 inhibitor RMC-4550 (30mg/kg, SHP2i).
B) Probability of survival for indicated treatment groups of the same experiment. Tumor growth for G12Ci (100 mg/kg RMC-4998) is shown in Figure 7F.
C) Bioluminescence scans of co-engrafted tumors with a subpopulation of 0.04% Luc-eGFP KPAR.E2 cells by treatment group, indicating the relative abundance of KPAR.E2 cells over time, measured in total flux, photons per second [p/s]. Mice were treated for 2 weeks (grey area) with the RAS-G12C(ON) inhibitor RMC-4998 (100 mg/kg; G12Ci) with or without the SHP2 inhibitor RMC-4550 (30 mg/kg; SHP2i) or a-PD-1 (10 mg/kg).
D) Mouse weights from mice in panel C during the treatment period with the combination therapies. Each dot represents a single measurement, and connected dots track individual mice over time.
