# Supplementary material for: KRAS Inhibitor–Induced Cancer Cell Death Enhances Sensitivity to Immune-Mediated Bystander Killing of Drug-Resistant Subclones
Source: Cancer Res. 2026 Jun 8;86(17):4334–51. doi: 10.1158/0008-5472.CAN-25-5693 (PMC13535323; doi:10.1158/0008-5472.CAN-25-5693)
Supplement: Supplementary file 7 [file can-25-5693_figure_s1_suppsf1.pdf]

Supplementary Figure S1

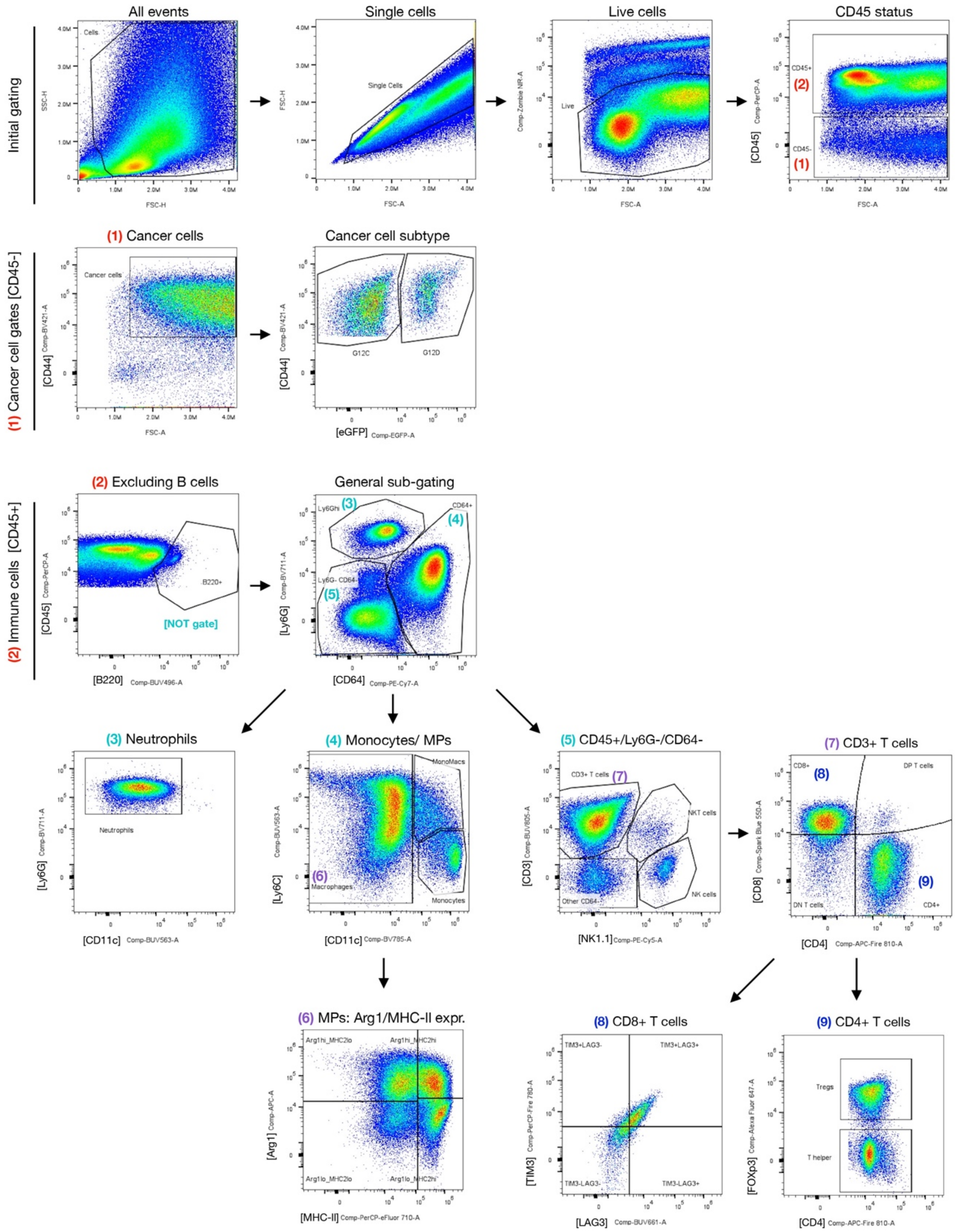

Supplementary Figure S1. Spectral flow cytometry gating strategy.
Gating strategy from flow cytometry experiment shown in Figure 5. Numbers on top of the graphs refer to the gate number in previous graphs and indicate the population that is plotted. Images from different tumors are shown to ensure sufficient representation of all indicated subpopulations for the gating strategy.
