# Supplementary material for: KRAS Inhibitor–Induced Cancer Cell Death Enhances Sensitivity to Immune-Mediated Bystander Killing of Drug-Resistant Subclones
Source: Cancer Res. 2026 Jun 8;86(17):4334–51. doi: 10.1158/0008-5472.CAN-25-5693 (PMC13535323; doi:10.1158/0008-5472.CAN-25-5693)
Supplement: Supplementary file 8 [file can-25-5693_figure_s2_suppsf2.pdf]

Supplementary Figure S2

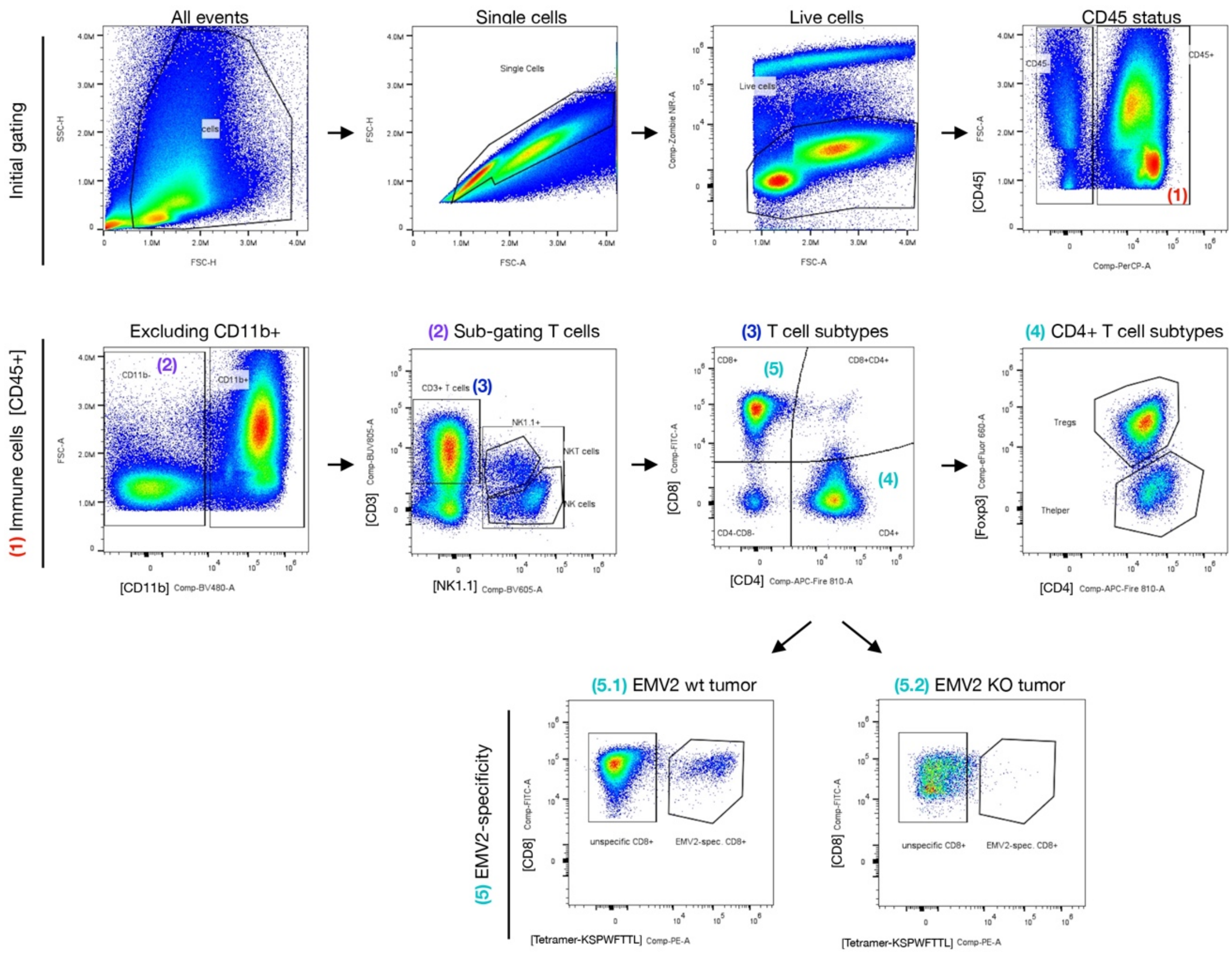

Supplementary Figure S2. Spectral flow cytometry gating strategy for Emv2 tetramer. Gating strategy from flow cytometry experiment to detect Emv2-specific CD8$^+$ T cells shown in Figure 6. Numbers on top of the graphs refer to the gate number in previous graphs and indicate the population that is plotted.
