# Supplementary material for: KRAS Inhibitor–Induced Cancer Cell Death Enhances Sensitivity to Immune-Mediated Bystander Killing of Drug-Resistant Subclones
Source: Cancer Res. 2026 Jun 8;86(17):4334–51. doi: 10.1158/0008-5472.CAN-25-5693 (PMC13535323; doi:10.1158/0008-5472.CAN-25-5693)
Supplement: Supplementary file 9 [file can-25-5693_figure_s3_suppsf3.pdf]

Supplementary Figure S3

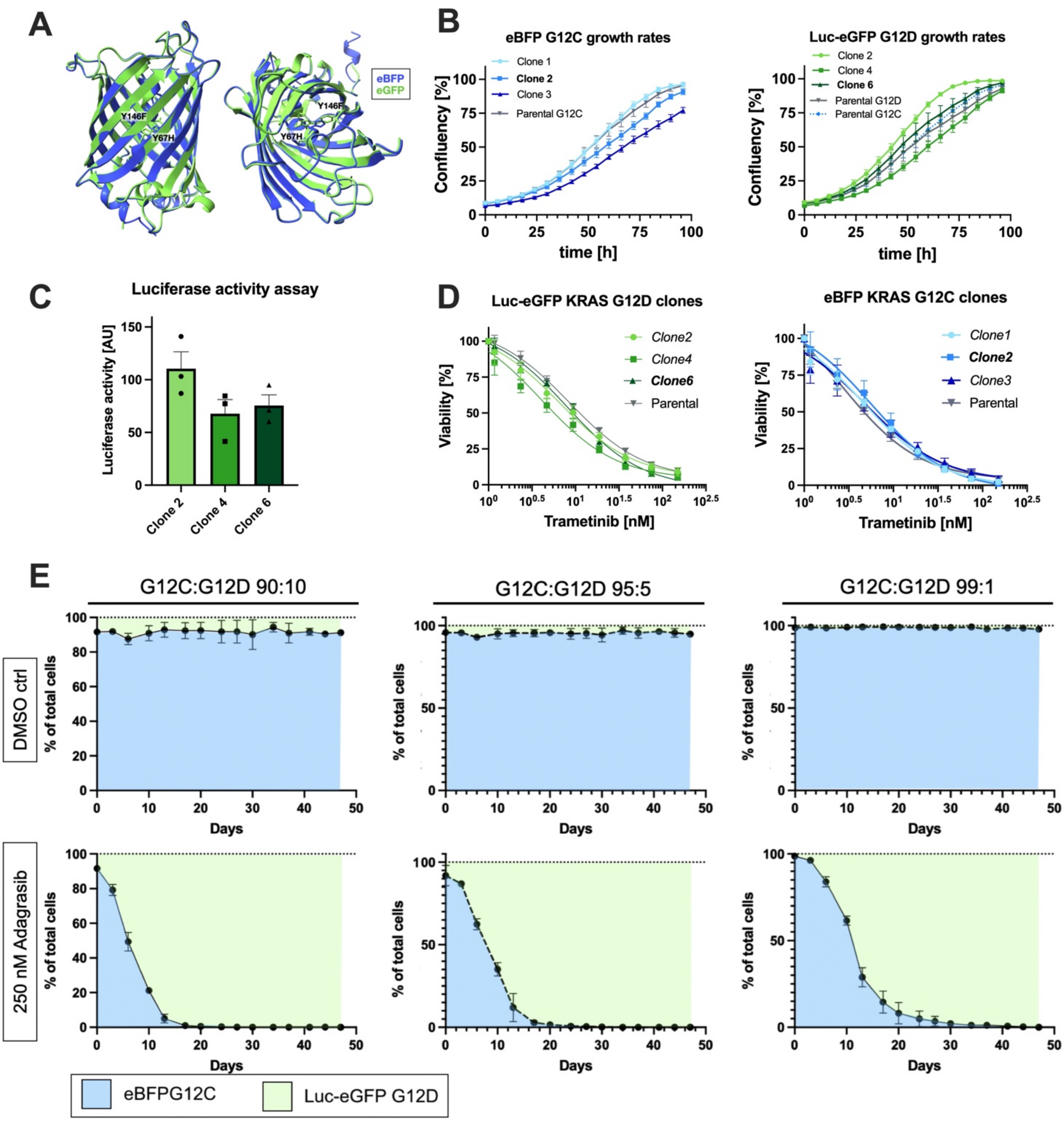

Supplementary Figure S3. Generation of reporter-transduced KPAR1.3 cell lines.
A) Prediction of structural similarity between eBFP and eGFP. eBFP protein structure is based on AlphaFold prediction, eGFP protein based on previously solved protein crystal structure (PDB: 4EUL), alignment based on the Needleman-Wunsch algorithm.
B) Representative plots of parental KPAR1.3 cells and selected Luc-eGFP-KRAS-G12D (left) or eBFP-KRAS-G12C (right) subclones, showing proliferation rates over time assessed via IncuCyte live imaging. n=3, mean ± SEM. For comparison purposes, right graph also contains the proliferation rate of the parental KPAR1.3 G12D cells.
C) Luciferase activity of KPAR1.3 Luc-eGFP-KRAS-G12D sub-clonal cell lines. Data is normalised to parental KPAR1.3 cells. n=3, tr(n) = 3, mean ± SEM.
D) Viability assays of selected subclones compared to parental counterparts. Cells were treated with the MEK inhibitor trametinib for 72 hours. n = 3, tr(n) = 3, mean ± SEM.
E) Co-culture assay assessing adagrasib response over time. Mixed cultures of eBFP-KRAS-G12C and Luc-eGFP-KRAS-G12D cells were continuously treated with DMSO or 250 nM adagrasib over time and fraction of G12C and G12D cells was measured by FACS at indicated time points. Blue and green areas on graphs depict the fractions of KPAR1.3 KRAS-G12C or -G12D populations, respectively. Mean ± SD; n = 3 to day 37; n = 2 to day 44, n = 1 to day 47, with 3 technical replicates in each round.
