# Supplementary material for: KRAS Inhibitor–Induced Cancer Cell Death Enhances Sensitivity to Immune-Mediated Bystander Killing of Drug-Resistant Subclones
Source: Cancer Res. 2026 Jun 8;86(17):4334–51. doi: 10.1158/0008-5472.CAN-25-5693 (PMC13535323; doi:10.1158/0008-5472.CAN-25-5693)
Supplement: Supplementary file 10 [file can-25-5693_figure_s4_suppsf4.pdf]

Supplementary Figure S4

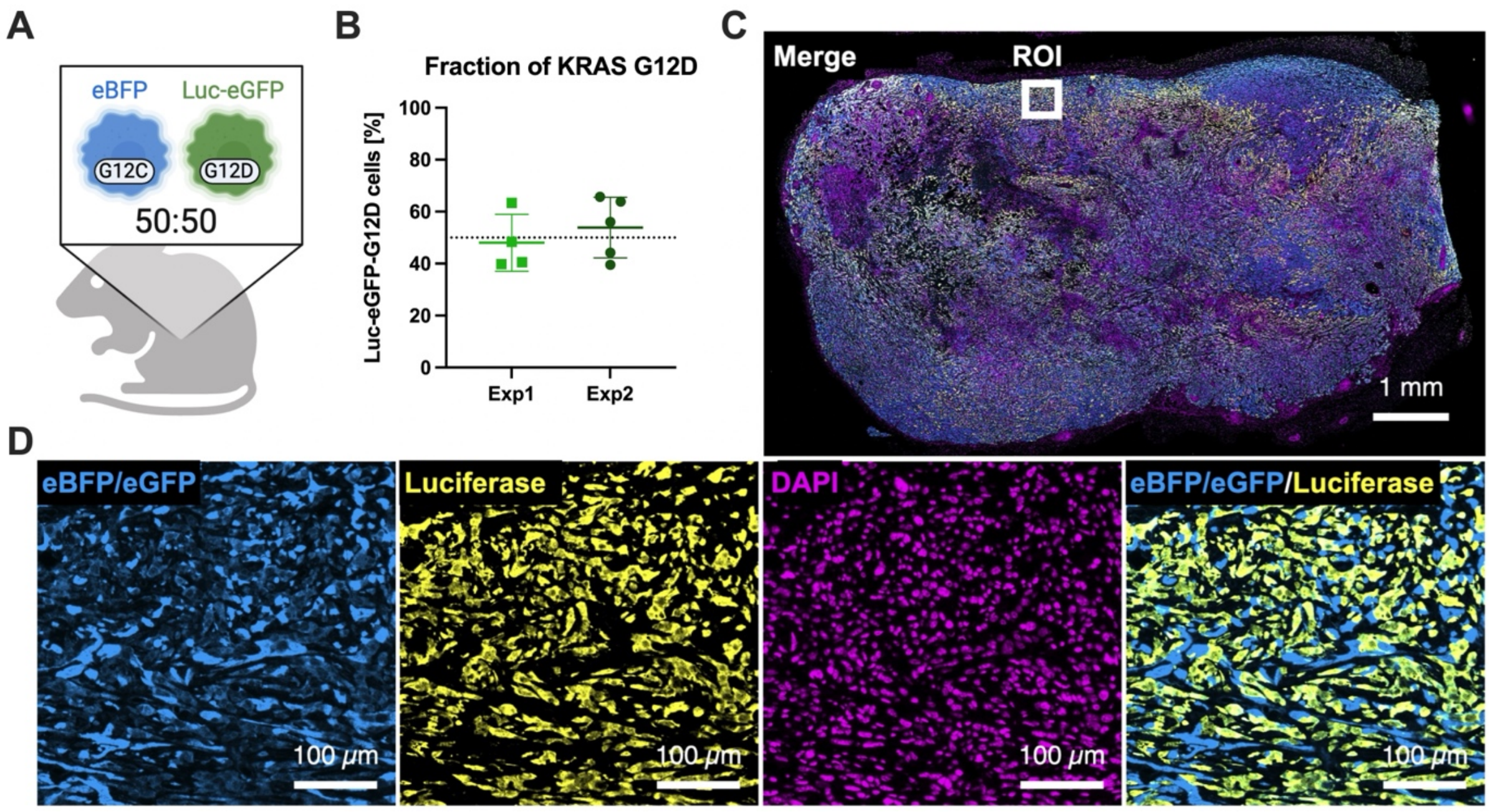

Supplementary Figure S4. Characterisation of mixed KPAR 1.3 tumors.

A) Schematic of experiment. Luc-eGFP-KRAS-G12D and eBFP-KRAS-G12C cells were co-engrafted subcutaneously in GH-mice at a 50:50 ratio to assess the growth dynamics of co-engrafted subclones in vivo.

B) Fraction of Luc-eGFP-KRAS-G12D cells within all live cancer cells of two independent experiments. Flow cytometric analysis was performed 14 days after engraftment of tumors. Dots represent individual tumors, with one subcutaneous tumor per mouse; mean ± SD.

C-D) Representative immunohistochemistry staining of one tumor 14 days after co-engraftment. C) Full tumor overview (scale bar 1mm) and D) Representative ROIs of individual channels (scale bar 100 μm). eBFP/eGFP: blue (G12C and G12D cells); luciferase: yellow (G12D cells); DAPI: magenta (nucleus).
