# Supplementary material for: KRAS Inhibitor–Induced Cancer Cell Death Enhances Sensitivity to Immune-Mediated Bystander Killing of Drug-Resistant Subclones
Source: Cancer Res. 2026 Jun 8;86(17):4334–51. doi: 10.1158/0008-5472.CAN-25-5693 (PMC13535323; doi:10.1158/0008-5472.CAN-25-5693)
Supplement: Supplementary file 11 [file can-25-5693_figure_s5_suppsf5.pdf]

Supplementary Figure S5

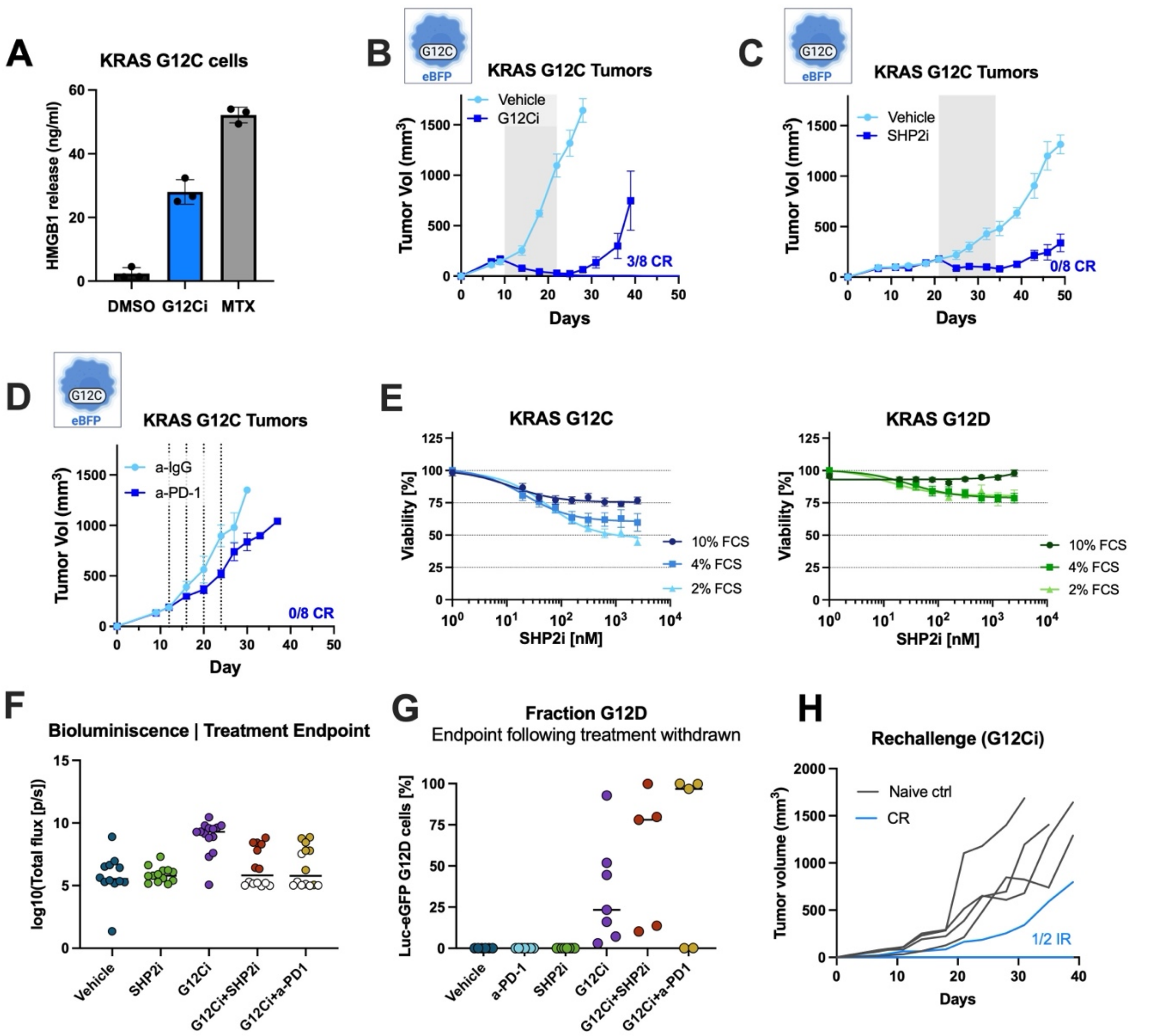

Supplementary Figure S5. Combination strategies to enhance treatment responses.
A) HGMB1 release in KPAR1.3 KRAS-G12C cells treated *in vitro* for 48 hours with DMSO control, 100 nM RMC-4998 (G12Ci) or 1 μM mitoxantrone (MTX). n=3, tr(n) = 2, mean ± SEM.
B) eBFP-KRAS-G12C subcutaneous tumors were treated daily for two weeks (grey area) with vehicle (n=6) or the RAS-G12C(ON) inhibitor RMC-4998 (100mg/kg; G12Ci; n=8). Number of complete responders (CR) is indicated. Mean ± SEM.
C) eBFP-KRAS-G12C subcutaneous tumors were treated daily for two weeks (grey area) with vehicle (n=8) or the SHP2 inhibitor RMC-4550 (30mg/kg; SHP2i; n=8). Mean ± SEM.
D) eBFP-KRAS-G12C subcutaneous tumors were treated with four doses (dotted lines) of isotype control (a-IgG, n=7) or a-PD-1 (10 mg/kg, n=8). Mean ± SEM.

E) Viability of eBFP-KRAS-G12C (left) and Luc-eGFP-KRAS-G12D (right) cells treated for 72 hours with serial dilutions of RMC-4550 (SHP2i). Cells were cultured with the indicated percentages of FCS. n=3-4, tr(n)=3, mean ± SEM.
F) Log10-transformed data of bioluminescence scans from mice in Figure 2C at treatment endpoint. Each dot represents one tumor; means are indicated by black lines; CR mice are highlighted in white across treatment groups.
G) Endpoint tumors from Figure 2C were isolated and fraction of Luc-eGFP-KRAS-G12D cells over all cancer cells was determined via flow cytometry. Each dot represents one tumor; means are indicated by black lines.
H) Naïve mice and two complete responders in (B) were reinjected with untraced KRAS-G12D KPAR1.3 cells on the opposite flank. Number of mice that immune rejected (IR) the re-injected cancer cells are indicated.
