# Supplementary material for: KRAS Inhibitor–Induced Cancer Cell Death Enhances Sensitivity to Immune-Mediated Bystander Killing of Drug-Resistant Subclones
Source: Cancer Res. 2026 Jun 8;86(17):4334–51. doi: 10.1158/0008-5472.CAN-25-5693 (PMC13535323; doi:10.1158/0008-5472.CAN-25-5693)
Supplement: Supplementary file 12 [file can-25-5693_figure_s6_suppsf6.pdf]

Supplementary Figure S6

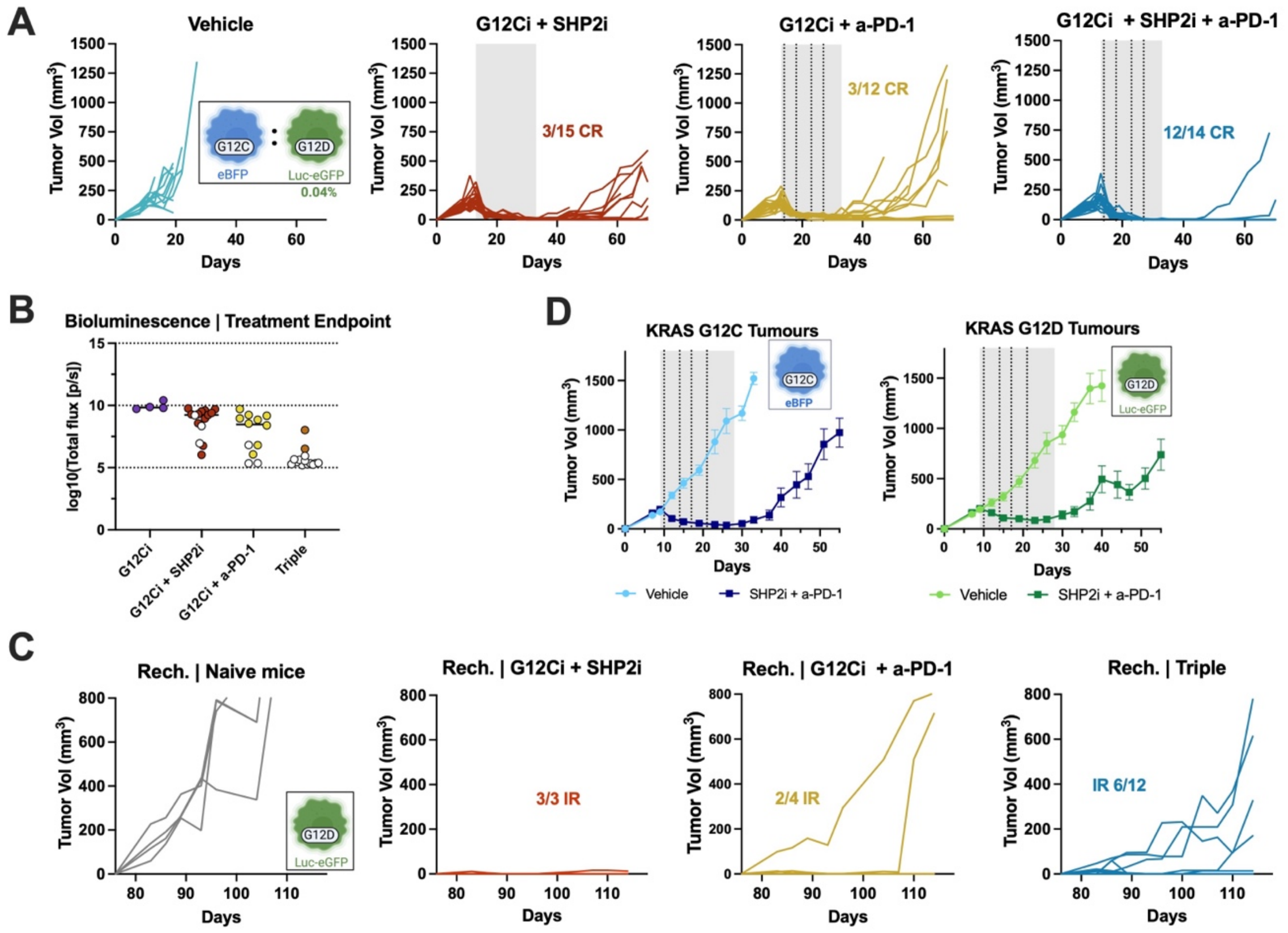

Supplementary Figure S6. Triple combination increases the generation of complete responders.

A) Tumor growth of mixed subcutaneous tumors engrafted with BFP-KRAS-G12C cells plus a fraction of 0.04% Luc-eGFP-KRAS-G12D cells. Mice were treated for three weeks with the RAS-G12C(ON) inhibitor RMC-4998 (100 mg/kg; G12Ci) with or without the SHP2 inhibitor RMC-4550 (30 mg/kg, SHP2i) and/or a-PD-1 (10 mg/kg). RMC-4998 and RMC-4550 were dosed by oral gavage daily (grey area) and a-PD1 was administered i.p. twice a week (vertical dotted lines). Complete responders (CR) are indicated.

B) Log10-transformed data of bioluminescence scans of mice in panel (A) at treatment endpoint by treatment groups. Each dot represents one tumor. Means are indicated by black vertical lines; CR mice are highlighted in white across treatment groups.

C) Naïve mice and complete responders in (A) were injected in the opposite flank with Luc-eGFP-KRAS-G12D cells. Mice that immune rejected (IR) the re-injected cancer cells are indicated. Graph titles indicate the treatment that the primary tumor received.

D) eBFP-KRAS-G12C (left) or Luc-eGFP-KRAS-G12D (right) subcutaneous tumors were treated for three weeks with either vehicle or a combination of RMC-4550 (30mg/kg; SHP2i) plus a-PD-1 (10mg/kg). N=12-13. Mean ± SEM.
