# Supplementary material for: KRAS Inhibitor–Induced Cancer Cell Death Enhances Sensitivity to Immune-Mediated Bystander Killing of Drug-Resistant Subclones
Source: Cancer Res. 2026 Jun 8;86(17):4334–51. doi: 10.1158/0008-5472.CAN-25-5693 (PMC13535323; doi:10.1158/0008-5472.CAN-25-5693)
Supplement: Supplementary file 13 [file can-25-5693_figure_s7_suppsf7.pdf]

Supplementary Figure S7

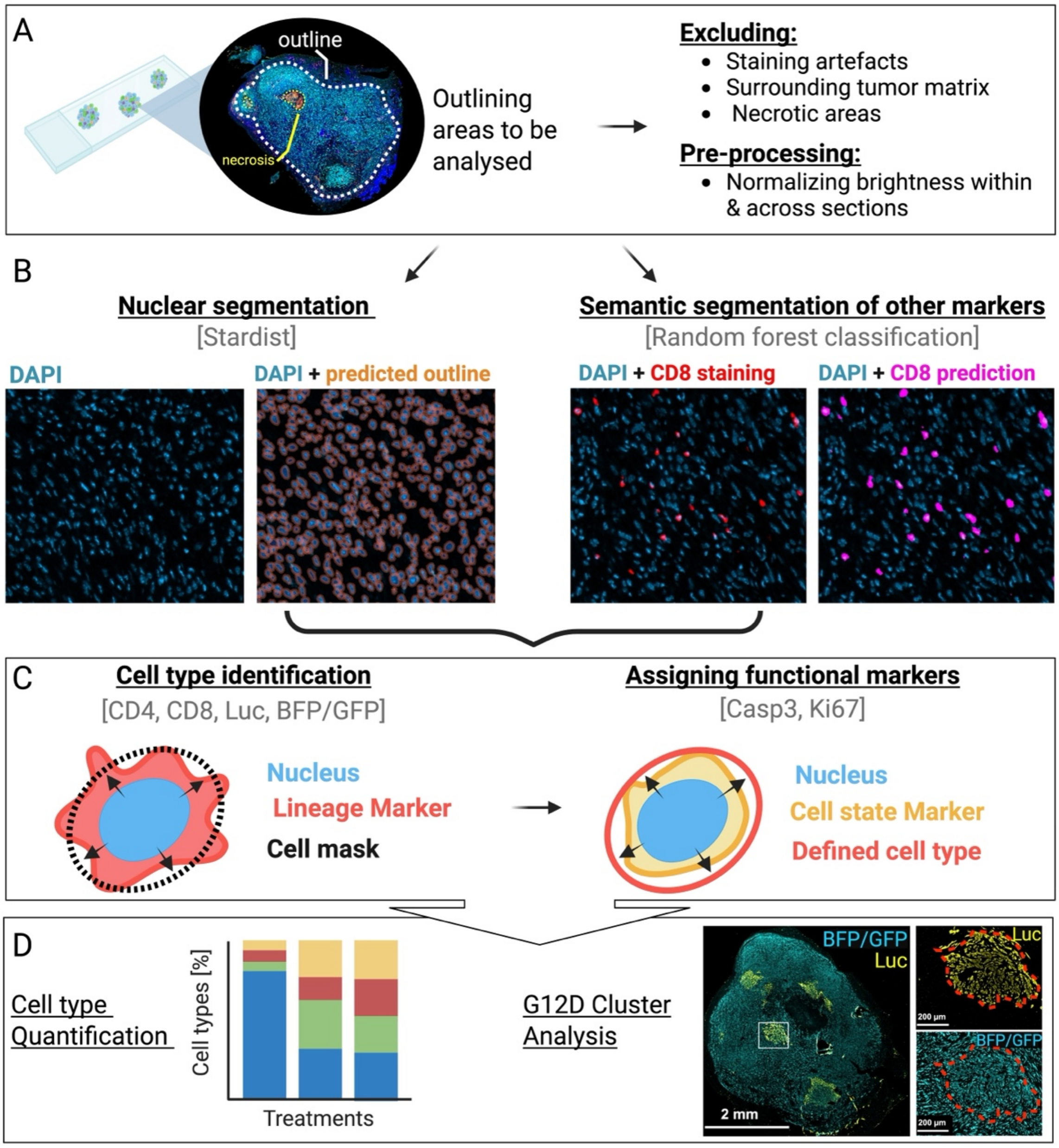

Supplementary Figure S7. Outline of image analysis pipeline.
A) Objective slide containing several tumor sections was imaged in one frame, ROIs within image were outlined (white outline) excluding staining artefacts and the matrix surrounding the tumor; necrotic areas were also demarcated as separate entities (yellow outlines); these areas were excluded for subsequent analysis.
B) Left panel: exemplary ROI showing nuclear segmentation, right panel: exemplary ROI showing semantic segmentation of $CD8^+$ T cells.
C) Schematic of subsequent steps. Left panel: cell type identification using the lineage markers of BFP/GBP and Luciferase for cancer cell as well as CD8 and CD4 to demarcate T cells. Right panel: Subsequently, defined cell types are assigned positive or negative values for the functional state markers Ki67 and Casp3.
D) After cell type classification, respective populations can be quantified.
