# Supplementary material for: KRAS Inhibitor–Induced Cancer Cell Death Enhances Sensitivity to Immune-Mediated Bystander Killing of Drug-Resistant Subclones
Source: Cancer Res. 2026 Jun 8;86(17):4334–51. doi: 10.1158/0008-5472.CAN-25-5693 (PMC13535323; doi:10.1158/0008-5472.CAN-25-5693)
Supplement: Supplementary file 14 [file can-25-5693_figure_s8_suppsf8.pdf]

Supplementary Figure S8

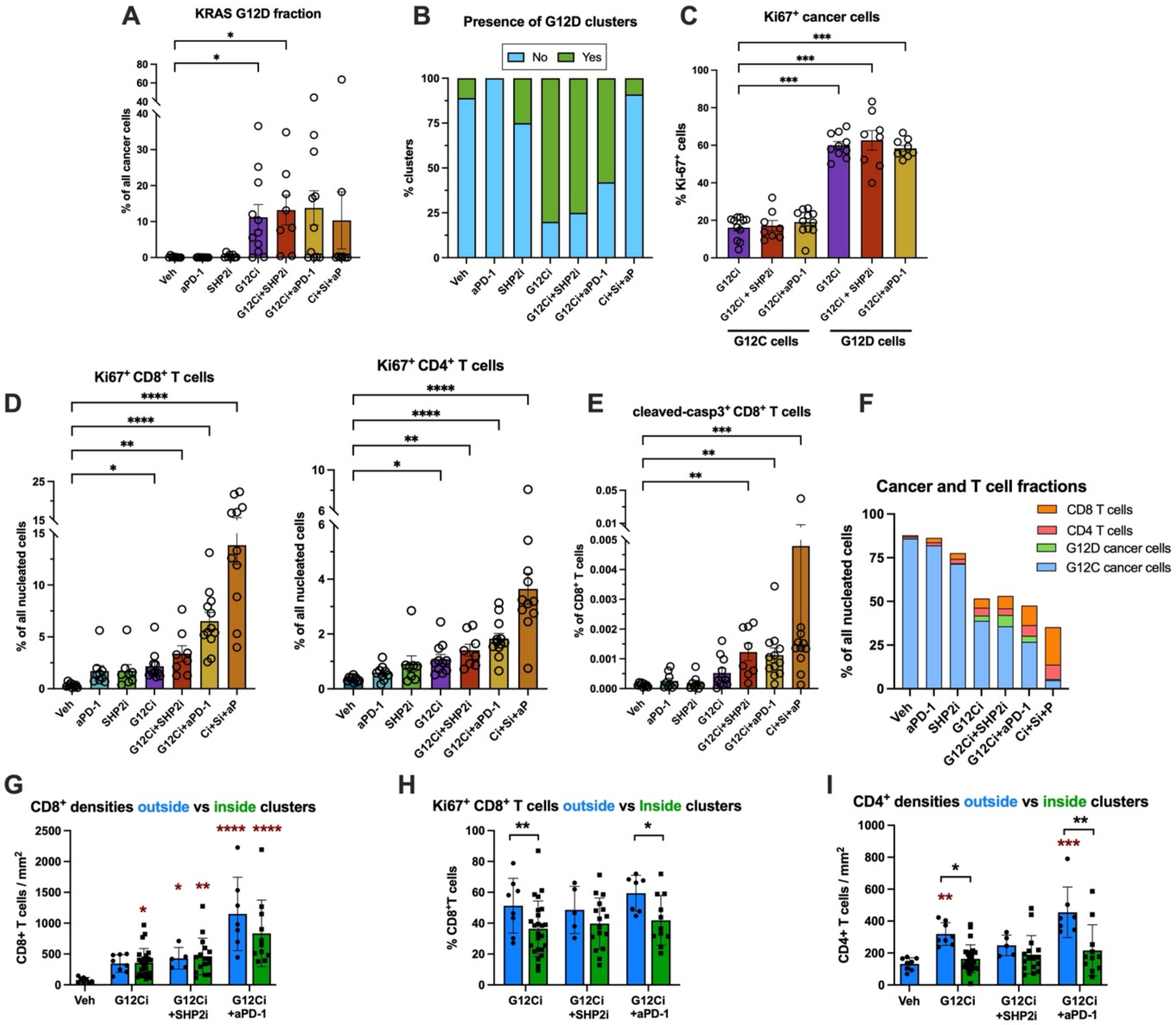

Supplementary Figure S8. Spatial analysis of mixed KPAR1.3 subcutaneous tumors. Mixed subcutaneous tumors were treated for six days with the RAS-G12C(ON) inhibitor RMC-4998 (100 mg/kg; G12Ci / Ci) with or without the SHP2 inhibitor RMC-4550 (30 mg/kg, SHP2i / Si) and/or a-PD-1 (10 mg/kg; P). RMC-4998 and RMC-4550 were dosed by oral gavage daily and a-PD-1 was administered i.p. twice a week. Tumors were analysed by multiple immunofluorescence as indicated in Figure 3A. Unless otherwise indicated dots represented individual tumors; one section per tumor was analysed (N=8-12). Statistics indicate one-way ANOVA test comparing each of the treatment groups to the vehicle; only significant comparisons are shown.

A) Fraction of KRAS-G12D mutant cells out of all cancer cells.

B) Percentages of tumor sections containing G12D clusters, defined as cell aggregates with more than 100 G12D cells.

C) Fraction of Ki67-positive KRAS-G12C and G12D cancer cells treated with G12Ci or the combinations.

D) Fraction of Ki67-positive $CD8^+$ and $CD4^+$ T cells out of all nucleated cells.
E) Fraction of cleaved-caspase 3 positive $CD8^+$ T cells out of all $CD8^+$ T cells.
F) Fraction of T cells and cancer cells of all nucleated cells. Data is plotted as average of all the tumor sections analysed (N=8-12). Individual tumor data are plotted in Figures 3B, 3C and 3G.
G-I) Quantification of G) $CD8^+$, H) Ki67-positive $CD8^+$ T cells and I) $CD4^+$ T cells in the G12D clusters (inside; green bars) compared with a representative section outside the cluster (blue bars). The dots in the blue bars show one representative section per tumor and the dots in the green bars correspond to all the clusters identified (defined in Figure 3D). Statistics in black indicate two-way ANOVA test comparing the inside and outside areas. For panels G and I, statistics in red indicate one-way ANOVA test comparing each of the treatment group to the vehicle. Only significant comparisons are shown.
