# Supplementary material for: KRAS Inhibitor–Induced Cancer Cell Death Enhances Sensitivity to Immune-Mediated Bystander Killing of Drug-Resistant Subclones
Source: Cancer Res. 2026 Jun 8;86(17):4334–51. doi: 10.1158/0008-5472.CAN-25-5693 (PMC13535323; doi:10.1158/0008-5472.CAN-25-5693)
Supplement: Supplementary file 15 [file can-25-5693_figure_s9_suppsf9.pdf]

Supplementary Figure S9

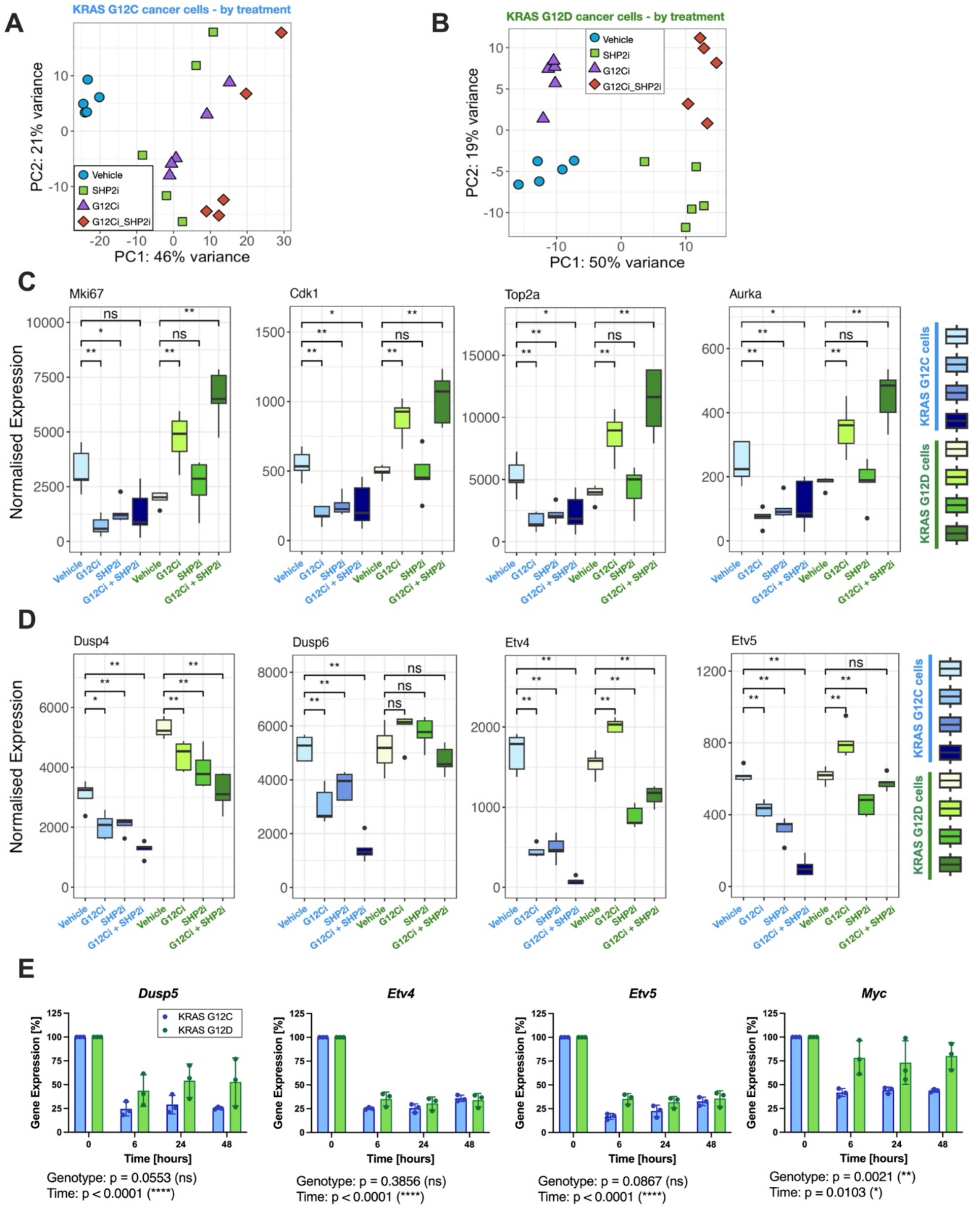

Supplementary Figure S9. RNAseq analysis of proliferation-associated tumor cell signatures.

Mixed subcutaneous tumors were engrafted with BFP-KRAS-G12C cells plus 7.5% Luc-eGFP-KRAS-G12D cells. Mice were treated for five days with the RAS-G12C(ON) inhibitor RMC-4998 (100 mg/kg; G12Ci) with or without the SHP2 inhibitor RMC-4550 (30 mg/kg; SHP2i). After treatment cancer cells were sorted and bulk RNAseq was performed.

(A-B) PCA analysis across all samples for A) KRAS-G12C and B) KRAS-G12D mutant tumor cell samples, with treatment groups indicated by shapes and colours. Each dot represents one independent biological replicate from a separate mouse.

(C-D) Expression of individual genes related to (C) proliferation and cell cycle progression and (D) KRAS downstream targets visualised as box plots indicating median, interquartile range. Whiskers represent the largest and smallest quartile and outliers are displayed as individual dots. Statistical significance between vehicle-treated and inhibitor-treated samples was assessed using pairwise Wilcoxon rank-sum tests (two-sideD).

E) qPCR analysis of transcripts for *Dusp5*, *Etv4*, *Etv5* and *Myc* in KRAS-G12C or -G12D KPAR1.3 cells treated in vitro (2% FBS) with 1 µM RMC-4550 (SHP2i) for indicated time points. Data was normalised to DMSO-treated cells. Mean ± SD, n=3, tr(n)=2. Two-way ANOVA test was performed to assess genotype (G12C vs G12D) and time differences.
